# Dipole-Programmed Chiral Porous Superlattice from Triangular Molecular Platforms on Ag(111)

**DOI:** 10.1021/acs.jpclett.6c02295

**Published:** 2026-08-27

**Authors:** Behzad Mortezapour, Sebastian Hamer, Rainer Herges, Hyeji Choi, Martin Wolf, Akitoshi Shiotari, Roberto Robles, Nicolás Lorente, Richard Berndt

**Affiliations:** † Institut für Experimentelle und Angewandte Physik, 234685 Christian-Albrechts-Universität zu Kiel, 24098 Kiel, Germany; ‡ Otto-Diels-Institut für Organische Chemie, Christian-Albrechts-Universität zu Kiel, 24098 Kiel, Germany; ¶ Department of Physical Chemistry, 28259 Fritz-Haber Institute of the Max-Planck Society, Faradayweg 4-6, 14195 Berlin, Germany; § Centro de Física de Materiales CFM/MPC (CSIC-UPV/EHU), 20018 Donostia-San Sebastián, Spain; ∥ Donostia International Physics Center (DIPC), 20018 Donostia-San Sebastián, Spain

## Abstract

Trioxatriangulenium was functionalized with a rotatable pyridyl moiety (pyridyl-TOTA), which exhibits a large electrostatic dipole moment, and investigated on the Ag(111) surface using low-temperature scanning tunneling microscopy (STM) and noncontact atomic force microscopy (NC-AFM). The molecules arrange themselves in a complex, well-ordered long-range pattern, namely, a chiral hexagonal mesh of rings composed of six molecules. Submolecularly resolved STM images reveal the orientations of the molecular dipoles, which are confirmed by NC-AFM measurements. Density functional theory (DFT) calculations reproduce the STM images and their intriguing dependence on the junction bias and confirm that the experimental results allow us to fully determine the orientation of the functional units. Comparison with phenyl-TOTA reveals that the dipole–dipole interaction of the pyridyl unit is strong enough to change the structure from a hexagonal close-packed pattern to a chiral hexagonal mesh of supramolecular hexagons. These results show that dipole functionalization may serve to control the formation of molecular assemblies on the surfaces.

## Graphical abstract

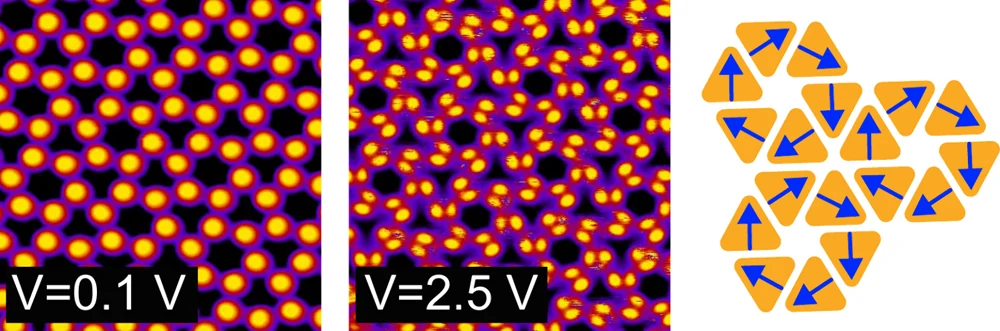

The self-assembly of molecules on single-crystal surfaces is intriguingly complex.^1–5^ Some simplification may be achieved by matching the symmetry of the molecules and the substrate. For example, molecules with the shape of an equilateral triangle have been studied on the hexagonal surface meshes provided by fcc(111) surfaces. In this case, the molecules often arrange themselves into honeycomb or hexagonal meshes,^6–13^ although striped patterns have also been reported.^14^

The present work deals with the triangular molecular platform trioxatriangulenium (TOTA) that can be functionalized with axial ligands.^15–22^ Typically, TOTA adsorbs planar on the substrate and forms hexagonal and honeycomb meshes.[Fn fn1] Here, we investigate the effect of a ligand with a strong electrical dipole moment. In 3-pyridyl-TOTA on Ag(111) (Figure 1), the N atom of the pyridyl subunit creates a significant dipole moment of 2.4 D according to density functional theory (DFT) calculations. Using scanning tunneling microscopy (STM) and noncontact atomic force microscopy (NC-AFM) we find that the molecules aggregate into huge islands with a new, highly ordered superstructure denoted spacious hexagonal that may be viewed as a hexagonal mesh of supramolecular hexagons. The TOTA pattern is chiral, and in addition, the pyridyl subunits orient themselves in a chiral fashion in each supermolecule. DFT calculations reproduce the STM images and their dependence on the sample voltage and show that the orientations of the ligands can be directly determined from the STM images. The calculations indicate that several competing interactions of similar magnitude control the assembly process. As a result, determining the optimal structure from DFT calculations is challenging.

**Figure 1:**
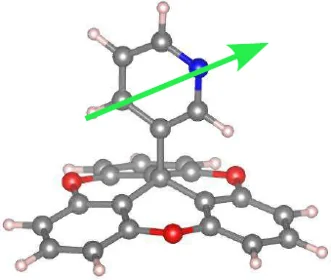
Scheme of 3-pyridyl trioxatriangulenium. The orientation of the molecular dipole moment is indicated by a green arrow.

A typical STM topograph of a submonolayer coverage of pyridyl-TOTA on Ag(111) is presented in Figure 2a. The area is comprised of two terraces (violet and orange) separated by a monatomic substrate step decorated with TOTA molecules. The larger terrace is covered by part of a large molecular island. The molecules are arranged in a regular pattern that is shown in more detail Figure 2b. A complex but regular pattern of circular protrusions is observed, each corresponding to a pyridyl-TOTA molecule. Each molecule has three nearest neighbors at an intermolecular distance of 0.95 ± 0.02 nm. The pattern can be constructed from building blocks of six molecules (encircled by dashed lines in Figure 2b), which in turn are arranged in a hexagonal mesh. A minimal unit cell with six molecules is indicated by a pink rhombus. Primitive vectors **a** and **b** are 2.73 ± 0.02 nm long and enclose an angle of 60°. The unit cell is rotated by 5 ± 2° (or −5° for an equivalent structure with the opposite chirality) compared to a close-packed direction of the Ag(111) substrate. A tentative model of the superstructure is presented in Figure 2c. It is derived from previous results for the self-assembly of TOTA derivatives on the (111) surfaces of Ag and Au. The molecules are located such that they form two hydrogen bonds with each of its three nearest neighbors. This bond pattern was previously proposed for methyl-TOTA on Au(111).^23^ We denote this molecular pattern as spacious hexagonal because it contains a regular array of pores. The pores occur in an alternating sequence of hexagonal and two triangular areas with the triangles being rotated by 180° with respect to each other.

**Figure 2:**
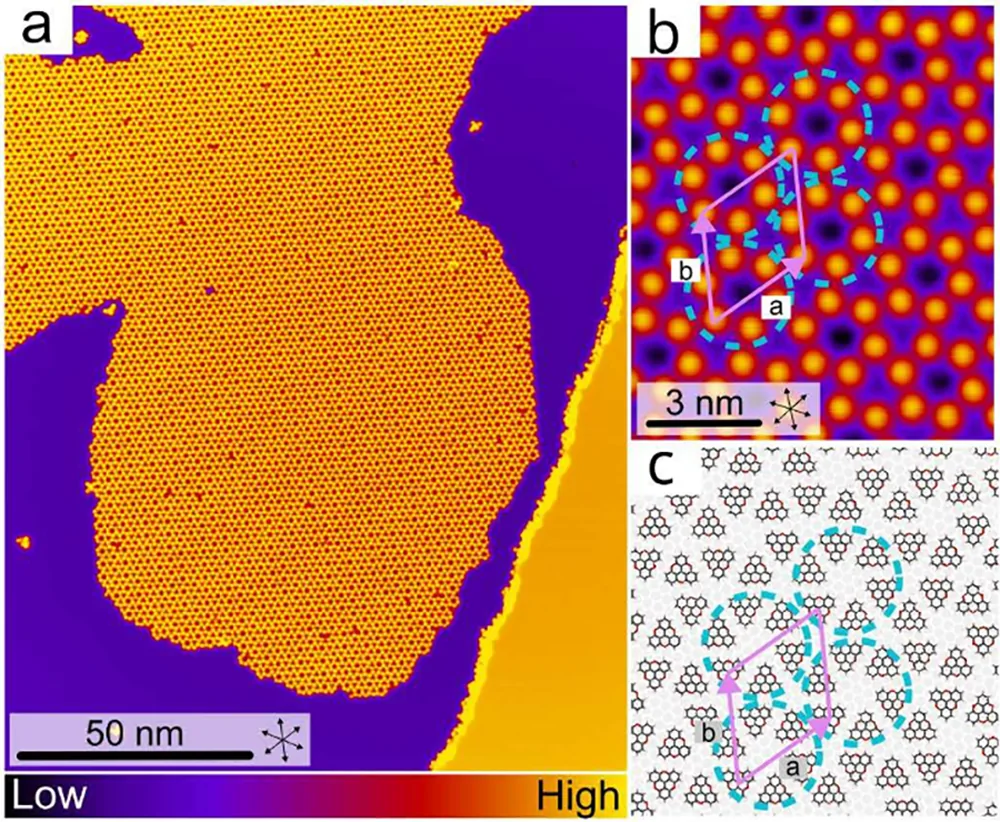
(a) Overview STM topograph (*V* = 0.5 V) of pyridyl-TOTA on Ag(111). Double-headed arrows indicate densely packed directions of the Ag(111) substrate. (b) Detailed topograph (0.5 V) of the interior of a pyridyl-TOTA island. A unit cell with primitive vectors **a** and **b** is indicated by a pink rhombus. Dashed circles show examples of hexagonal molecular rings. (c) Arrangement of the TOTA platforms suggested by the topographs. The ligands have been omitted for the sake of clarity. The unit cell is rotated by −5° with respect to a close-packed substrate direction.

Returning to Figure 2a, we find that the defect density in the island is low. Among ≈11 600 molecules, only ≈50 molecules appear lower, a value that is typical of all islands observed. Close inspection of the defects reveals a small ligand, presumably isopropyl that was used during the synthesis in a Grignard reaction. In addition, there is a tendency of the islands edges two be rather straight and oriented along the ⟨11̅0⟩ and ⟨112̅⟩ directions. Detailed images of these preferred step orientations are presented in the Supporting Information. Annealing of the surface to ≈45 °C for 2 h leads to the desorption of a large fraction of the molecules. While the remaining islands (Figure 3) have an approximately hexagonal shape, the molecular pattern is unaffected. To a large extent, the island edges are comprised of molecular hexagons and are oriented along ⟨11̅0⟩ ± 5°. A much smaller fraction of the edges is oriented along ⟨112̅⟩ ± 5°, where hexagons are bridged with three molecules. We rarely observed single molecules at the edges. These observations indicate that hexagons may serve as building blocks during the growth of the pattern.

**Figure 3:**
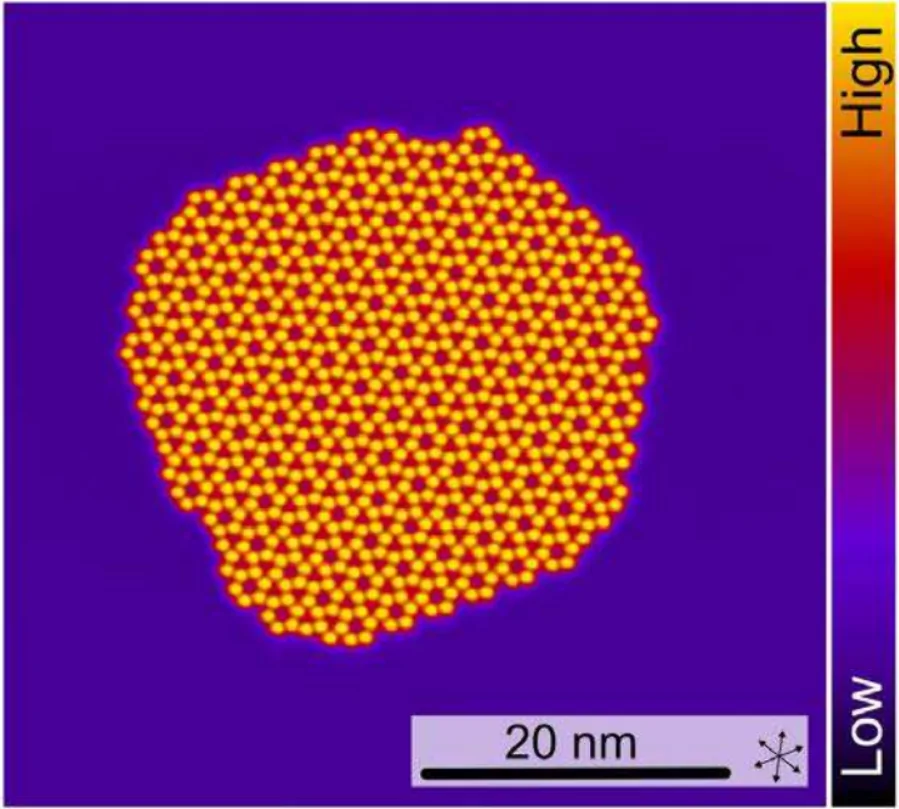
STM topograph of a pyridyl-TOTA island recorded after annealing. Double-headed arrows indicate densely packed directions of the Ag(111) substrate.

While the spacious hexagonal mesh was vastly predominant, we also observed a hexagonal close-packed pattern on rare occasions. Corresponding data are displayed in the Supporting Information.

At negative (down to −2.5 V) and low positive (up to ≈1.5 V) sample voltages, the molecules appear as circular protrusions in constant current topographs (Figure 4a,b). However, more increased positive bias leads to additional structures. In 2.1 V data, Figure 4c, the protrusions are elliptical. The orientations of the long axes change by 60° between neighbor molecules in hexagonal rings. At a further increased voltage (*V* = 2.5 V) (Figure 4d), a nodal line is resolved along the short axis of each ellipse. Similar results from phenyl-TOTA suggest that the nodal line reveals the orientations of the pyridyl subunits. Closer inspection shows that the long axes of the resulting narrow ellipses are not strictly parallel. We figuratively denote them as arrowheads, which, intriguingly, form a head-to-tail configuration along the circumference of the hexagonal molecular rings. The orientation of the arrowheads is linked to the chirality of the molecular pattern. The chirality in Figure 4 always exhibits a clockwise arrangement of the arrowheads (see Figure 4d), while the chirality in Figure 5b corresponds to a counterclockwise arrangement.

**Figure 4:**
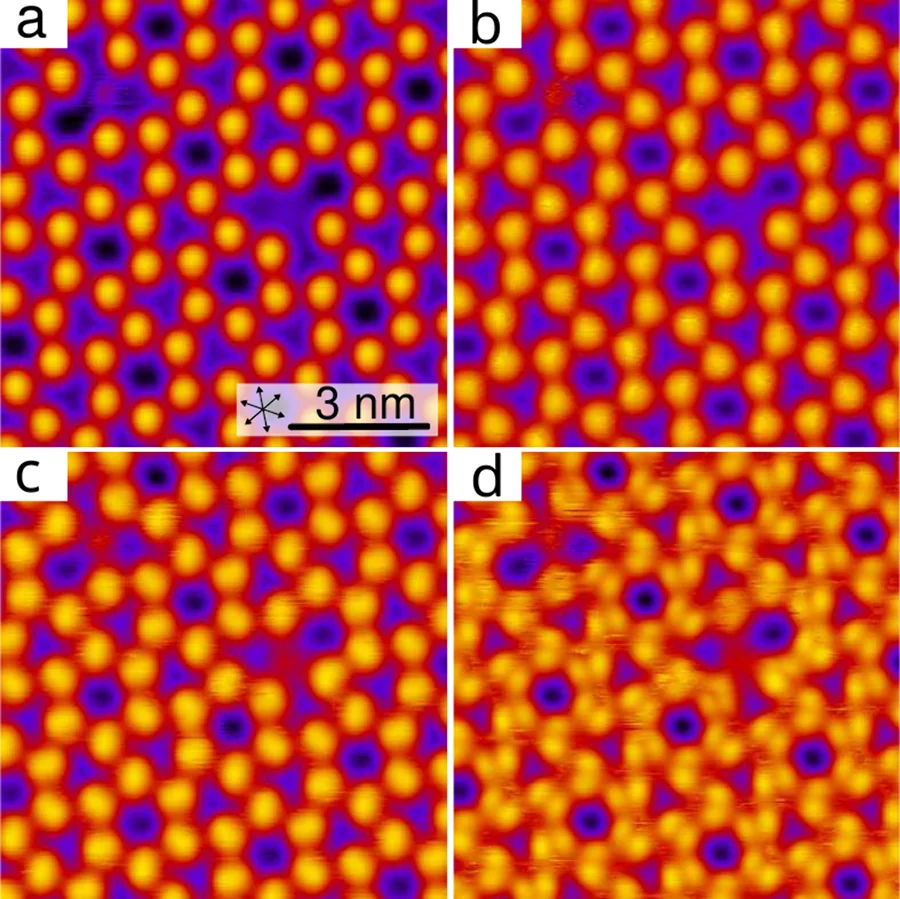
STM topographs of a pyridyl-TOTA island recorded at different sample voltages. (a) When *V* = 0.5 V, the molecules appear as circular protrusions. (b) When *V* = −2.5 V, the image contrast is hardly changed. (c) At an increased positive voltage of 2.1 V, the molecules give rise to an elliptical protrusion. (d) At 2.5 V, a nodal line is discernible in each molecule. In addition, the separation between the resulting two parts varies along the direction of the nodal line.

**Figure 5:**
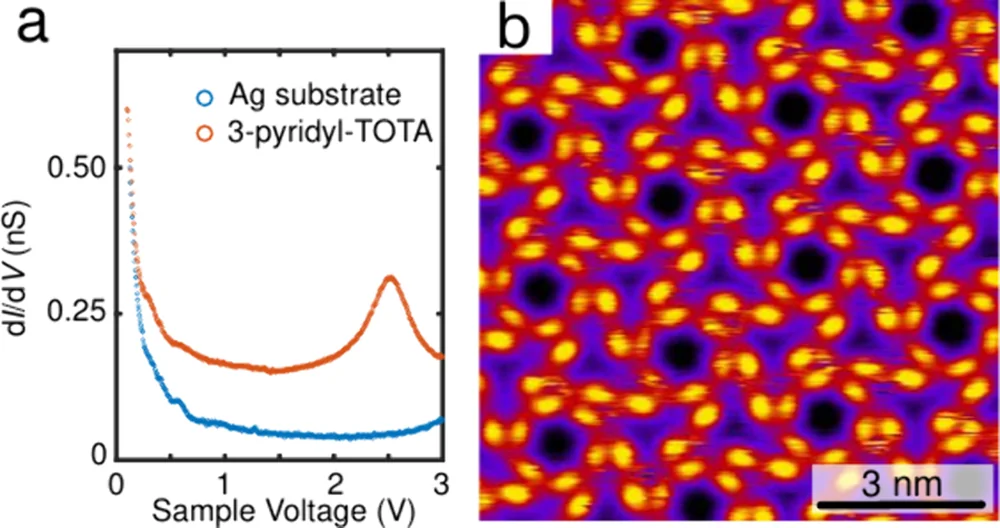
Spectroscopic data. (a) Constant-current d*I*/d*V* spectra obtained above the center of an ellipse as observed at 2.1 V, and above the substrate, recorded at *I* = 50 pA. The molecular spectrum (red curve, vertically shifted for the sake of clarity) exhibits a resonance centered near 2.5 V that is not observed on the substrate (blue curve). (b) Constant-height d*I*/d*V* map recorded at 2.5 V with feedback disabled at *I* = 50 pA. Each molecule gives rise to two slightly diverging elliptical protrusions. The chirality of the structure displayed here was used in the calculations and is the opposite of that of Figure 4.

We further investigated the electronic structure of the molecular assembly by spectroscopy of the differential conductance d*I*/d*V* of the molecules (Figure 5a), the results being consistent with the voltage-dependent changes in the contrast in the topographs. While the spectrum of the Ag(111) substrate (blue line) is nearly featureless, the pyridyl-TOTA spectrum exhibits a pronounced peak centered at around 2.5 V. A map of the differential conductance recorded at the peak voltage and at a fixed tip height is displayed in Figure 5b. This map resembles the 2.5 V topography, indicating that the image contrast at this voltage is predominantly due to the unoccupied state near 2.5 eV. A tentative structural model taking the experimental observations into account is shown in Figure 6.

**Figure 6:**
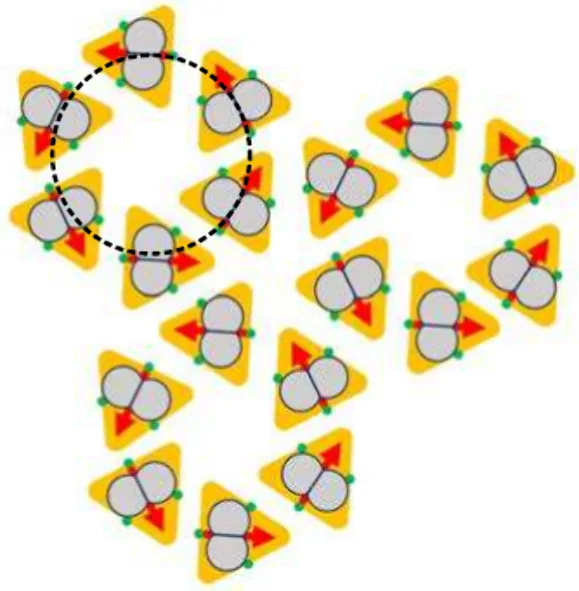
Tentative model of the pyridyl orientation. Green dots represent O atoms of the TOTA platform (yellow triangle). The orientations of the pyridyl moieties are symbolized by red arrows and schematic π-orbitals (gray).

To further verify the orientations of the ligands, we conducted NC-AFM imaging of pyridyl-TOTA on Ag(111) with a tip functionalized by carbon monoxide (CO), since CO tips can provide high spatial resolution in frequency shift (*Δf*) images.^24^ Figure 7a shows a typical image of a pyridyl-TOTA island, where the molecular center is imaged as a round bright spot with a dark halo. The appearance is analogous with NC-AFM images of other steric molecules with CO tips;^25–27^ the bright spot corresponds to the strong Pauli repulsion between the CO molecule and the protruding atoms of the pyridyl moiety, while the underlying TOTA platform provides a weak van der Waals (vdW) attractive interaction with the tip, resulting in the shallow halo. This image clearly characterizes a vacancy in a hexagonal molecular ring (top-right corner of the yellow frame in Figure 7a), but the orientation of the pyridyl moieties is barely visible in the image because the CO tip is highly sensitive to the topmost atoms (C–H) of the molecule.

**Figure 7:**
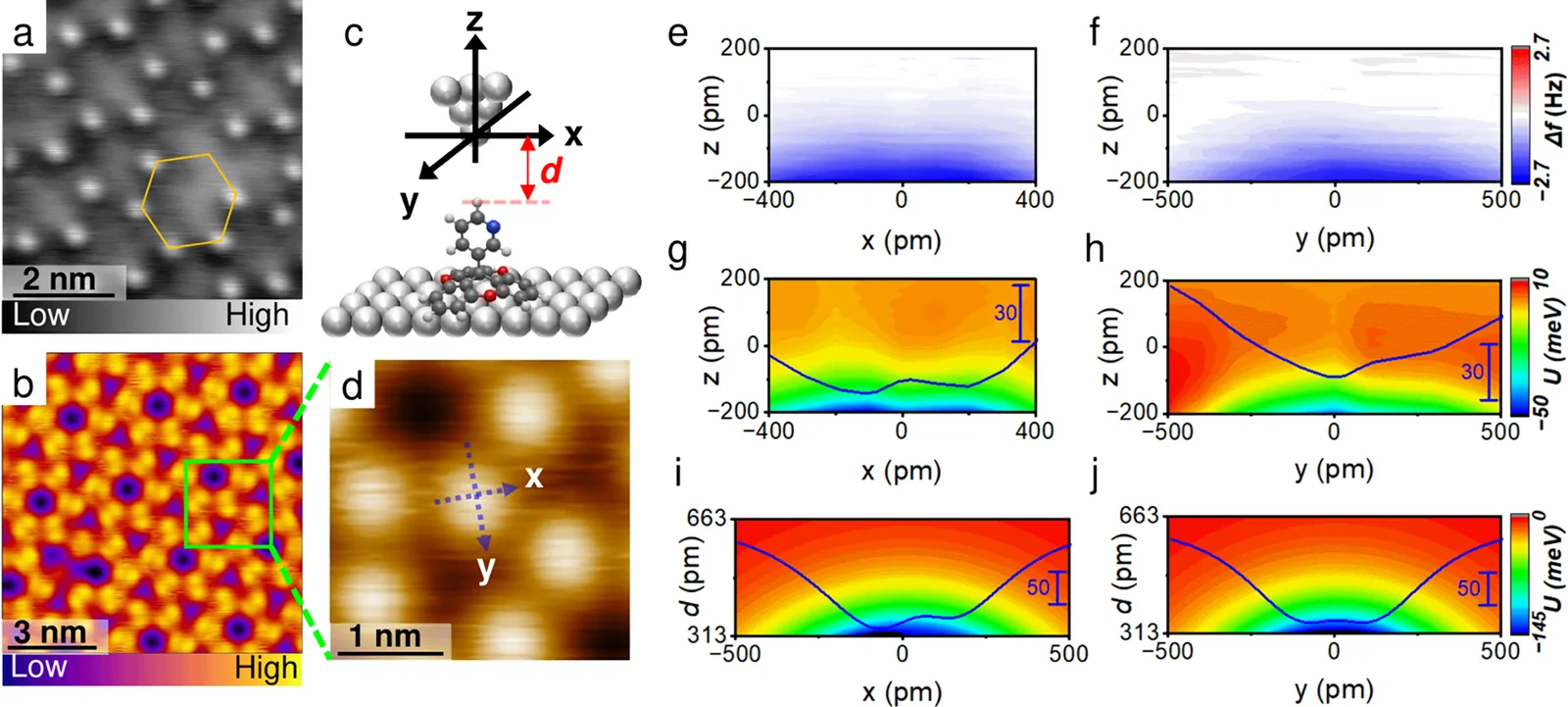
(a) NC-AFM image of pyridyl-TOTA/Ag(111) with a CO tip (*z* = 10 pm; *Δf* = −13.2 Hz (low) to −8.0 Hz (high)). A yellow hexagon indicates a hexagonal molecular ring where one molecule at a top-right corner of the hexagon is missing. (b) High-bias STM image (*V* = 2.5 V) with a metal tip. (c) Birds-eye view illustration of the junction between a metal tip and a pyridyl-TOTA molecule on Ag(111) for AFM mapping. (d) Low-bias STM image (*V* = 0.5 V) magnifying the square-framed area in panel b. (e and f) *Δf* maps in the *x*–*z* plane at *y* = 0 and in the *y*–*z* plane at *x* = 0, respectively, recorded over the molecule shown in panel d. (g and h) *U* maps in the *x*–*z* and *y*–*z* planes, respectively, obtained from the experimental *Δf* maps. (i and j) Simulated *U* maps with the lateral axes parallel and perpendicular, respectively, to the pyridyl ring plane. The vertical axis *d* indicates the distance between the topmost H atom of the molecule and the tip-apex atom. The overlaid blue curves in panels g–j represent the horizontal line profiles at the minimum tip height in each map.

To identify the pyridyl-group orientation by NC-AFM, we next conducted two-dimensional AFM mapping (also known as force mapping)^28–31^ using a metal tip. Figure 7d shows the measured molecule. In a high-bias STM image (Figure 7b), the same molecule was observed as a twin elliptical protrusion with a nodal line. The twin protrusion is asymmetric as the right-side nodal gap is wider, in a manner similar to that of the STM images shown in Figure 4d. Based on the node of the twin protrusion, we designate *x* and *y* axes as parallel and perpendicular, respectively, to the nodal line, as depicted by arrows in Figure 7d. Panels e and f of Figure 7 show *Δf* maps in the *x*–*z* and *y*–*z* planes, respectively. At any position, *Δf* decreases monotonically with a decrease in *z*, indicating attractive interactions between the molecule and the metal tip.^29^ Note that a smaller tip height (*z* < −200 pm) than used in the *Δf* map often caused vertical manipulation of the molecule to the tip.

From the *Δf* maps, we obtained force maps (see the Supporting Information) and potential energy maps (Figure 7g,h)^32^ to quantify the interaction between the tip and pyridyl-TOTA. The observed potential energy range of *U* > −50 meV implies the predominant contribution from vdW forces. Notably, the maps exhibit anisotropy in the *x* and *y* directions; the *U*(*x*, *z*) map has a double minimum at *x* ≈ ±150 pm, whereas *U*(*y*, *z*) has a minimum at the molecular center (see the line profile at *z* = −200 pm overlaid on each map). Moreover, the attractive area in the *x* direction is more broadly distributed than that along the *y* axis (yellow and green areas in each map). These features imply that the broader *U*(*x*, *z*) distribution results from contributions of multiple atoms of the molecules, while narrower *U*(*y*, *z*) originates from a single-atom thickness. Therefore, we attribute the *x* and *y* axes to the directions parallel and perpendicular, respectively, to the pyridyl ring plane, as shown in Figure 7c, consistent with the tentative model based on STM (Figure 6).

To support the assignment described above, we conducted potential map simulations using a simple model, where the tip was approximated as a single atom (namely, a probe particle^33^) and the pairwise Leonard-Jones potentials between the tip atom and each atom of the pyridyl group were summed to obtain *U* values^29–31^ (see the Supporting Information). The simulated maps (Figure 7i,j) show anisotropy in the directions parallel (*x*) and perpendicular (*y*) to the pyridyl plane, reasonably reproducing the experimental maps. The asymmetric N atom location (*x* > 0) (Figure 7c) results in the asymmetric double minimum potential with the N atom side weaker (see the line profile in Figure 7i) in the simulations. The experimental *x* direction map (Figure 7g) exhibits similar asymmetry with respect to *x*. However, this minute difference (<5 meV) may be caused by an asymmetric tip structure, and the simple simulation model ignores the contributions from the molecular dipole moment, electrostatic forces, and force-induced molecular deformation. Considering the chiral pattern of the high-bias STM images, nevertheless, the model shown in Figure 6 is the most plausible. Chiral molecular hexagons have recently also been observed from benzimidazole arrays on Ag(111).^34^

For comparison with an extended alkyl ligand of the TOTA platform, we briefly studied *sec*-butyl-TOTA (Figure 8a) on the same substrate. The lateral extensions of this ligand and pyridyl are similar. However, *sec*-butyl-TOTA has no dipole moment and also lacks the conjugated π-electron system that leads to interactions between pyridyl-TOTA molecules.^22^ As expected, the *sec*-butyl-TOTA molecules arrange themselves in large hexagonal islands (Figure 8a). The unit cell corresponds to 1.04 ± 0.03 nm superstructure and is identical to those previously observed from methyl-TOTA and phenyl-TOTA on a Ag(111) surface.^22,23^ At higher magnification (Figure 8b), the ligands are resolved as elliptical protrusions. This image contrast does not significantly change in the range from −2.5 to 2.5 V. Three distinct orientations are observed, but no indication of the order of the azimuthal ligand orientations was found. By bringing the tip close to a ligand, its orientation may be changed (Supporting Information).

**Figure 8:**
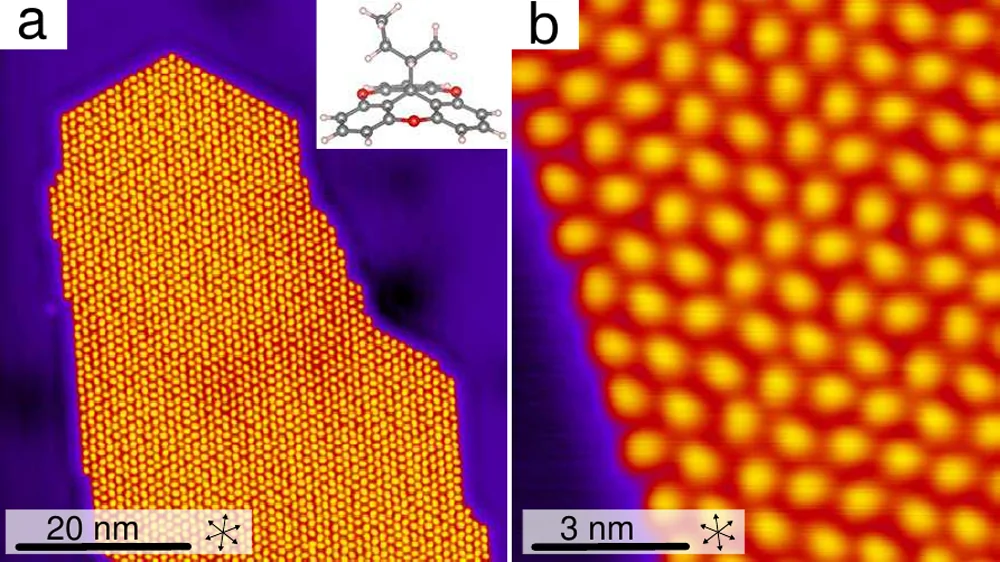
(a) Constant-current topograph (0.13 V) of a large monolayer island of *sec*-butyl-TOTA on Ag(111). A model is shown in the inset. (b) Detailed topograph (0.25 V) revealing the orientations of the ligands.

In order to theoretically study the spacious hexagonal arrangement derived from the experimental data, we performed a series of DFT calculations using the unit cell determined by the STM images. The best structure consistent with the experimental results is depicted in Figure 9a. Each molecule has three molecular neighbors, and it forms two hydrogen bonds with each of them.

**Figure 9:**
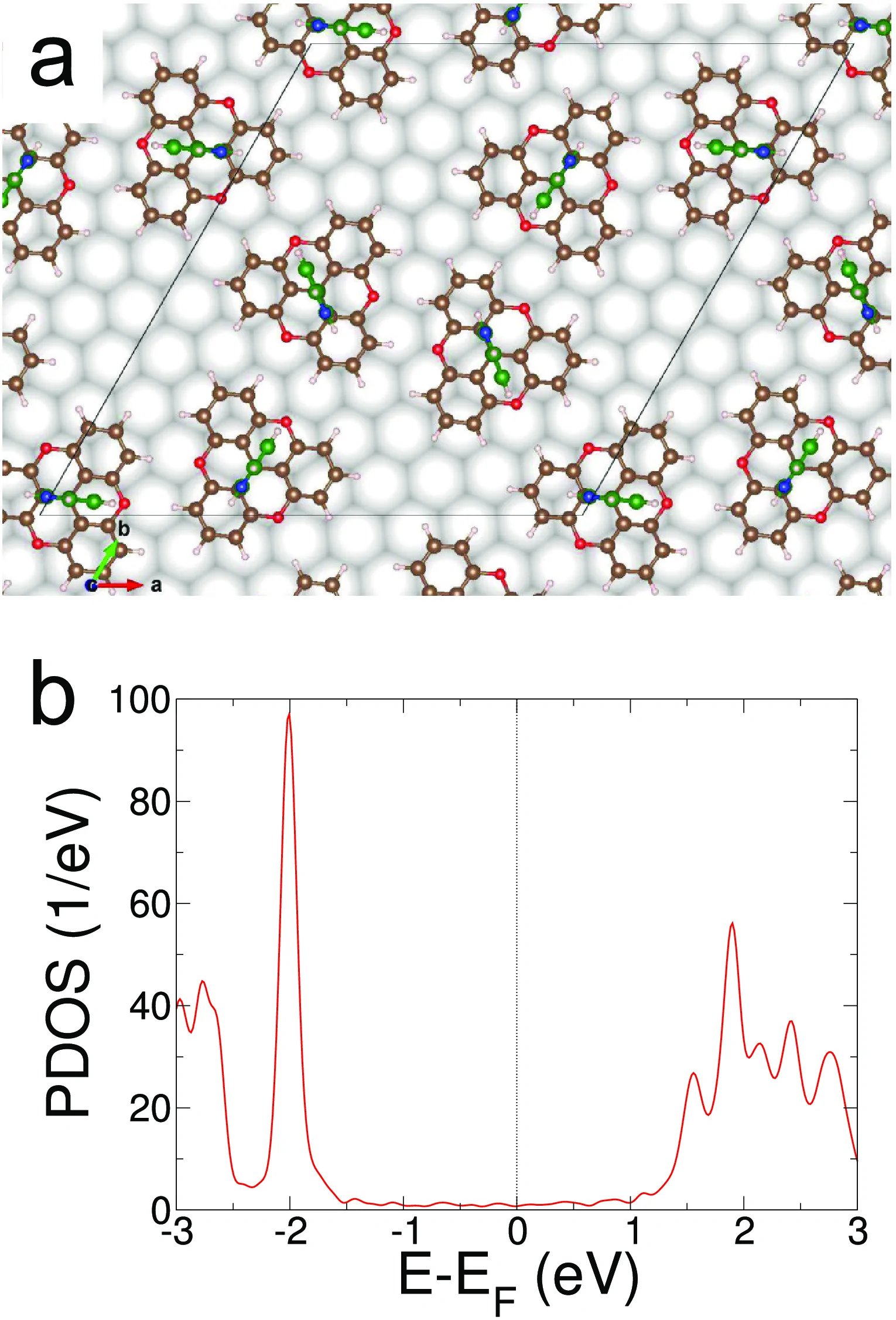
(a) Top view of the relaxed structure of the spacious hexagonal arrangement formed by six pyridyl-TOTA molecules on Ag(111). White, brown, red, blue, and pink spheres represent Ag, C, O, N, and H atoms, respectively. Green spheres represent C atoms of the pyridyl moiety. Black lines show the unit cell. (b) Density of states projected on pyridyl-TOTA molecules.

Simulated STM images are shown in Figure 10. They nicely reproduce the experimental topographs and their variation with the sample voltage used. At a low voltage (*V* = ±0.5 V (Figure 10a,b)), the molecules appear as roundish protrusions. This voltage is within the gap between the highest occupied and lowest unoccupied molecular orbitals (HOMO and LUMO, respectively), as one can see in the projected density of states (PDOS) shown in Figure 9b. At the onset of the first unoccupied state (*V* = 1.3 V), the molecules present a clear node in the middle (Figure 10c). The overlaid molecular structure shows that the nodal plane corresponds to the plane of the pyridyl moiety. Finally, a d*I*/d*V* map at 2.14 V is shown in Figure 10d. It also reproduces the experimental conductance map in Figure 5b, including the “arrowheads” pointing in a counterclockwise direction. By comparing the image with the molecular structure (see the inset), one can determine that the open end of the arrows indicates the position of the N atom, which is pointing away from an O atom in the TOTA platform. For an extended discussion of the origin of the arrows, see the Supporting Information.

**Figure 10:**
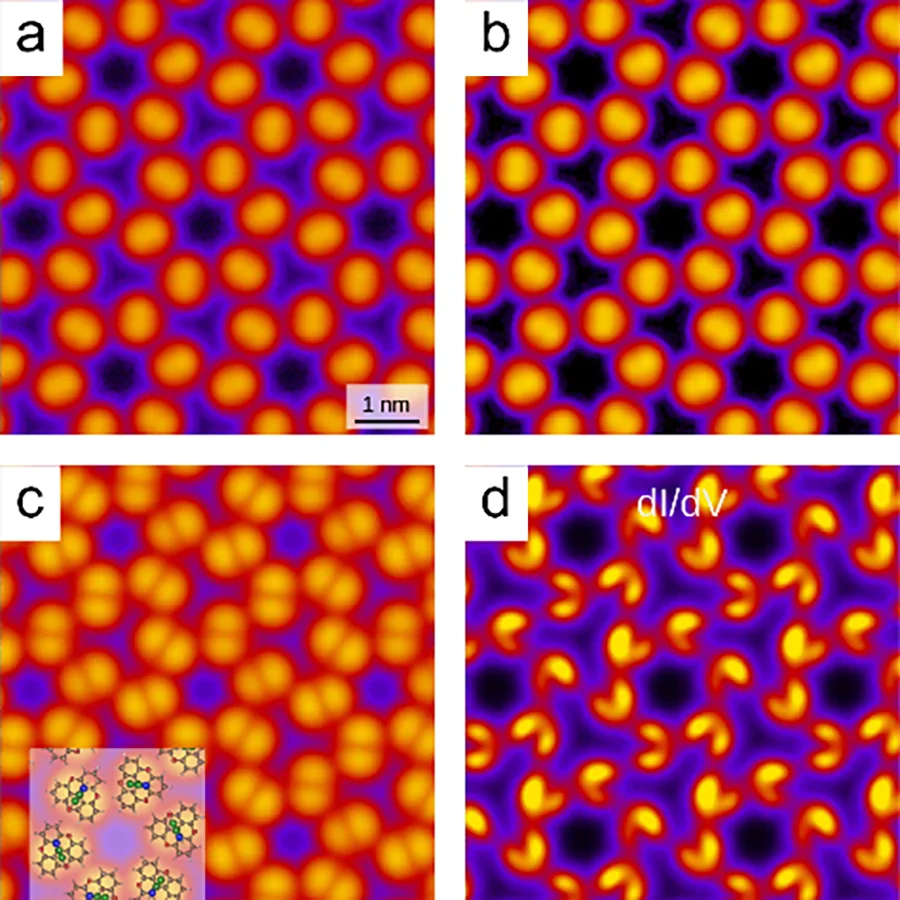
Simulated STM data of the structure in Figure 9a. Calculated constant-current STM images at *V* values of (a) 0.5, (b) −0.5, and (c) 1.3 V. (d) Calculated constant-height d*I*/d*V* map at *V* = 2.14 V.

A comparison between experimental and simulated data enables the determination of the full structure of the system, the only exception being the adsorption position. (i) The arrangement of the TOTA platforms is determined from the STM topographs at low voltages. (ii) The azimuthal orientation of the pyridyl units is determined by the nodal plane at higher voltages. (iii) The position of the N atom is determined by the “arrowhead” shape, mainly in the d*I*/d*V* maps.

The binding energy in the spacious hexagonal arrangement is 2.165 eV/molecule. The vdW binding energy, i.e., the binding energy calculated only with the vdW energies, is 2.506 eV/molecule. The rest of the binding energy is repulsive by −0.341 eV/molecule. This result is similar to the phenyl-TOTA molecule^22^ and is due to the vdW interaction that binds the molecules to the surface but also forces them to sit in an unfavorable position if only the non-vdW forces are taken into account. For an isolated adsorbed molecule, the binding energy is 2.063 eV (see the Supporting Information). In this case, vdW bonding occurs only between the molecule and the Ag substrate. Therefore, the difference with the binding energy of the spacious hexagonal arrangement, i.e., 103 meV/molecule, corresponds to intermolecular binding. If we just consider this layer in the gas phase, without a surface, the binding energy is 232 meV/molecule. Again, the vdW interaction binds the molecules to the surface but at the same time tends to destabilize the intermolecular binding.

We also studied the hexagonal close-packed arrangement found for phenyl-TOTA.^22^ The calculated binding energy in the present case of pyridyl-TOTA is 2.229 eV/molecule. This is close to the value of 2.238 eV/molecule obtained for phenyl-TOTA on Ag(111).^22^ The binding energy for the close-packed structure is 63 meV higher than that for the spacious one. This indicates a more stable situation, in contrast to the experimental results. For phenyl-TOTA, the energy difference between both structures is almost the same (62 meV/molecule). In this case, the structure observed experimentally is the hexagonal close-packed, in agreement with the theoretical prediction.

There are a few possible reasons for the discrepancy between the predicted lower-energy structure and the observed one. First, there are some technical limitations to the calculations. We approximate the crystal surface by a slab, and in this case, due to the large lateral size of the unit cell, we used only four Ag layers in the slab. When we increased the size of the unit cell by using a nine-layer slab, the energy difference between the structures decreased from 63 to 20 meV; i.e., the results came closer to the experimental situation. According to previous studies, nine layers already provide a proper description of the surface.^35^

Other possible reasons for the discrepancy are the kinetic effects, which may prevent the formation of the minimum-energy structure in the experiments. A factor that may play a role is the difference in binding motifs between the compact and spacious structures. The former involves corner-to-side hydrogen bonds, while the latter is based on side-to-side bonding (see the Supporting Information). Side-to-side bonding involves two C–H···O hydrogen bonds, and it is 28 meV more favorable in dimers. Therefore, it enhances the stability of hexagonal rings, which are formed by side-to-side bonding. Such rings were indeed observed from various TOTA derivatives on the (111) surfaces of Ag and Au.^22^ In large islands, however, the coordination number of each molecule is doubled in corner-to-side bonded hexagonal meshes, which increases the binding energy per molecule. It seems likely that side-to-side bonded clusters, in particular, hexagonal rings, form during the nucleation of the pyridyl-TOTA arrays. Their conversion to a dense hexagonal layer requires the breaking of this favorable side-to-side conformation. A subtle difference in the energy barrier for breaking the hexagonal rings may also be the reason why the spacious pattern is much more abundant than the compact mesh for pyridyl-TOTA. We have checked whether annealing of the spacious structure can overcome the barrier and lead the formation of the compact one. However, annealing of the surface to ≈45 °C for 2 h, while leading to the evaporation of a large fraction of the molecules (Figure 3), did not change the molecular pattern. The resulting islands are formed mostly by hexagons, confirming that this motif can work as a building block of the spacious structure.

The different behavior of pyridyl-TOTA and phenyl-TOTA^22^ can be rationalized by examining the different interactions at play. The TOTA platforms are identical in both cases and so are the interactions between them, mainly C–H···O bonds and vdW forces. The difference comes from the functional units. Phenyl and pyridyl are similar, the fundamental difference being the dipole moment of the second. The functional units are separated by a distance of ≈1 nm. Although this separation seems fairly large, direct long-range interactions between phenyl ligands have been found to determine the azimuthal orientation of the phyenyl subunit.^22^ Similar interactions are likely at play in the case of biphenyl-TOTA.^36^ The phenyl–phenyl interaction in the most favorable configuration (T-shaped) is around 4 meV. The extra interaction for pyridyl, namely the dipole–dipole interaction, is most favorable in a head-to-tail orientation and is about 5 meV for a 2.4 D dipole at an angle of 30°. Therefore, two competing interactions play a role in the kinetics leading to the formation of the observed structures.

In summary, we showed that the introduction of a molecular dipole into a TOTA platform is sufficient to modify its supramolecular assembly on a metal surface. Pyridyl-TOTA on Ag(111) does not adopt the hexagonal array previously found for closely related nonpolar analogues but forms a well-ordered, chiral hexagonal array of hexagonal clusters covering large surface areas. This structural transition demonstrates that modest changes in the electrostatic character of the functional group can lead to stabilization of qualitatively different molecular arrangements.

By combining low-temperature STM, NC-AFM, and DFT calculations, we identified the arrangement of the TOTA platforms, the azimuthal orientation of the pyridyl units, and the correspondence between the high-bias STM contrast and dipolar ligand orientation. This combined approach reveals that the self-assembled layer exhibits a well-defined orientational organization of the dipolar subunits within a chiral supramolecular motif.

The comparison with similar systems lacking an electric dipole like phenyl-TOTA and *sec*-butyl-TOTA highlights the central role of the pyridyl functionality: the emergence of the spacious hexagonal arrangements arises from the interplay between dipolar interactions, hydrogen bonding, and vdW stabilization on the surface. Although DFT identifies the hexagonal close-packed phase as energetically competitive or even slightly favorable, the experimentally observed spacious structure dominates, suggesting that the monolayer structure may be governed by thermodynamics and kinetic effects may be involved due to the stability of the hexagonal molecular building blocks.

More broadly, our results identify the functionalization with molecular dipoles as a viable strategy for engineering surface-supported molecular self-assembly in two-dimensional supramolecular materials. These results therefore hint at a general design concept for creating self-assembled two-dimensional structures in which weak, long-range intermolecular interactions govern the emergence of ordered metastable phases.

## Experimental and Computational Details

Experiments were carried out with a scanning tunneling microscope operated at ≈4.6 K in ultrahigh vacuum. Ag(111) surfaces were prepared by cycles of Ar^+^ sputtering with an ion energy of 1.5 keV and annealing to ≈500 °C. Pyridyl-TOTA molecules were synthesized as described in the Supporting Information. Pyridyl-TOTA and *sec*-butyl-TOTA were sublimated from crucibles heated to ≈65 °C onto the substrate at ambient temperature. STM tips were electrochemically etched from W wire and bombarded with Ar^+^ *in vacuo*. STM topographs were recorded at *I* ∼ 10 pA. The image dimensions were calibrated using atomically resolved images of the Ag substrate. Bias voltage *V* was applied to the sample. Spectroscopy of the differential conductance d*I*/d*V* was performed using a lock-in amplifier and adding a 10 mV_RMS_ modulation at frequencies between 410 and 685 Hz to *V*.

The NC-AFM experiments were carried out with another instrument (CreaTec Fischer & Co. GmbH) operated at 6 K in ultrahigh vacuum. The pyridyl-TOTA/Ag(111) sample was prepared in the same manner as described in the STM experiments. The sample at 10 K was exposed to CO gas, and a CO molecule adsorbed on the surface was picked up by a metal tip to yield a CO tip for NC-AFM imaging.^24^ We used a qPlus sensor with a W tip (spring constant of 1800 N/m, *Q* value of ∼10^4^, resonance frequency of 26.2 kHz, cantilever oscillation amplitude of 100 pm), enabling simultaneous operation of STM and frequency-modulation AFM. The NC-AFM image was obtained in constant-height mode^24^ with a CO tip (*V* = 0 mV), whereas the STM images were obtained in constant current mode (*I* = 20 pA during the cantilever oscillation). The origins of *x*, *y*, and *z* were determined by the tip position when the tip is placed over the center of the pyridyl-TOTA molecule with the STM feedback closed (during cantilever oscillation, *V* = 0.5 V and *I* = 20 pA). Two-dimentional AFM mapping was conducted with a metal tip (*V* = 0 mV) by recording a series of *Δf*(*z*) curves along the *x* or *y* direction. As a background curve, a *Δf*(*z*) curve was recorded over a central pore of the hexagonal molecular ring, where no short-range interaction between the tip and the pyridyl-TOTA molecules is expected. The raw *Δf*(*z*) curves over the molecule were subtracted by the background curve and plotted in the maps. The *z*-dependent force and potential at each (*x*, *y*) position were calculated from the background-subtracted *Δf*(*z*) curves using the Sader–Jarvis formula.^32^

Simulations were performed in the framework of density functional theory using the VASP code.^37^ Core electrons were treated using the projector-augmented-wave method,^38^ and wave functions were expanded using a plane wave basis set with an energy cutoff of 500 eV. Exchange and correlation were treated using the PBE functional^39^ complemented with the D3 method^40,41^ to treat dispersion interactions.

A Ag(111) surface was simulated using a slab with four layers of Ag and the experimental in-plane unit cell accommodating six molecules, making a total of 610 atoms in the unit cell. Additional calculations for selected cases were performed with a slab containing nine layers of Ag and a total of 1065 atoms. The vacuum region has at least 18 Å. Due to the size of the unit cell, the Γ point was enough to sample the Brillouin zone. A 4 × 4 × 1 *k*-grid was used to plot PDOS and to simulate STM images and d*I*/d*V* maps. Geometries of all atoms except the two bottom Ag layers were relaxed until forces were smaller than 0.01 eV/Å. Corrections to the potential and forces were applied to account for the presence of a dipole moment in a periodic slab.^42^

Adsorption energies *E*_ad_ per adsorbed molecule were calculated using

$$E_{\mathrm{ad}}\left[n\cdot \left(\mathrm{pyridyl‐}\mathrm{TOTA}\right)@\mathrm{Ag}\left(111\right)\right]=[n\cdot E\left[\mathrm{pyridyl‐}\mathrm{TOTA}\right]+E\left[\mathrm{Ag}\left(111\right)\right]-E\left[n\cdot \left(\mathrm{pyridyl‐}\mathrm{TOTA}\right)@\mathrm{Ag}\left(111\right)\right]/n$$

where *n* is the number of adsorbed molecules, *E*[pyridyl-TOTA] is the energy of one molecule, *E*[Ag­(111)] is the energy of the Ag(111) surface, and *E*[*n*·(pyridyl-TOTA)@Ag­(111)] is the energy of the full system.

STM images were simulated with the STMpw code,^43^ which uses the Tersoff–Hamann approximation^44^ within the method of Bocquet et al.^45^ Atomic and density plots were generated using VESTA.^46^

## Supplementary Material



## Footnotes

a Ligands that are larger than the TOTA platform itself are exceptions to this rule.^16^

## Notes

Further STM images of pyridyl-TOTA islands, additional spectroscopic data, hexagonally close-packed islands, NC-AFM data showing force maps and the method of potential energy map simulations, STM images of *sec*-butyl-TOTA on Ag(111), and additional theoretical data showing different configurations, d*I*/d*V* maps, and results for a single molecule (PDF)
