# Supplementary material for: Dipole-Programmed Chiral Porous Superlattice from Triangular Molecular Platforms on Ag(111)
Source: J Phys Chem Lett. 2026 Aug 27;17(36):10388–96. doi: 10.1021/acs.jpclett.6c02295 (PMC13573615; doi:10.1021/acs.jpclett.6c02295)
Supplement: Supplementary file 1 [file jz6c02295_si_001.pdf]

Dipole-Programmed Chiral Porous Superlattice from Triangular Molecular Platforms on Ag(111)

Behzad Mortezapour,[1] Sebastian Hamer,[2] Rainer Herges,[2] Hyeji Choi,[3] Martin Wolf,[3] Akitoshi Shiotari,[3,*] Roberto Robles,[4,†] Nicolás Lorente,[4,5] and Richard Berndt[1,‡]

[1] *Institut für Experimentelle und Angewandte Physik, Christian-Albrechts-Universität zu Kiel, 24098 Kiel, Germany*
[2] *Otto-Diels-Institut für Organische Chemie, Christian-Albrechts-Universität zu Kiel, 24098 Kiel, Germany*
[3] *Department of Physical Chemistry, Fritz-Haber Institute of the Max-Planck Society, Faradayweg 4-6, 14195 Berlin, Germany*
[4] *Centro de Física de Materiales CFM/MPC (CSIC-UPV/EHU), 20018 Donostia-San Sebastián, Spain*
[5] *Donostia International Physics Center (DIPC), 20018 Donostia-San Sebastián, Spain*

(Dated: August 25, 2026)

Edges of 3-pyridyl-TOTA Islands

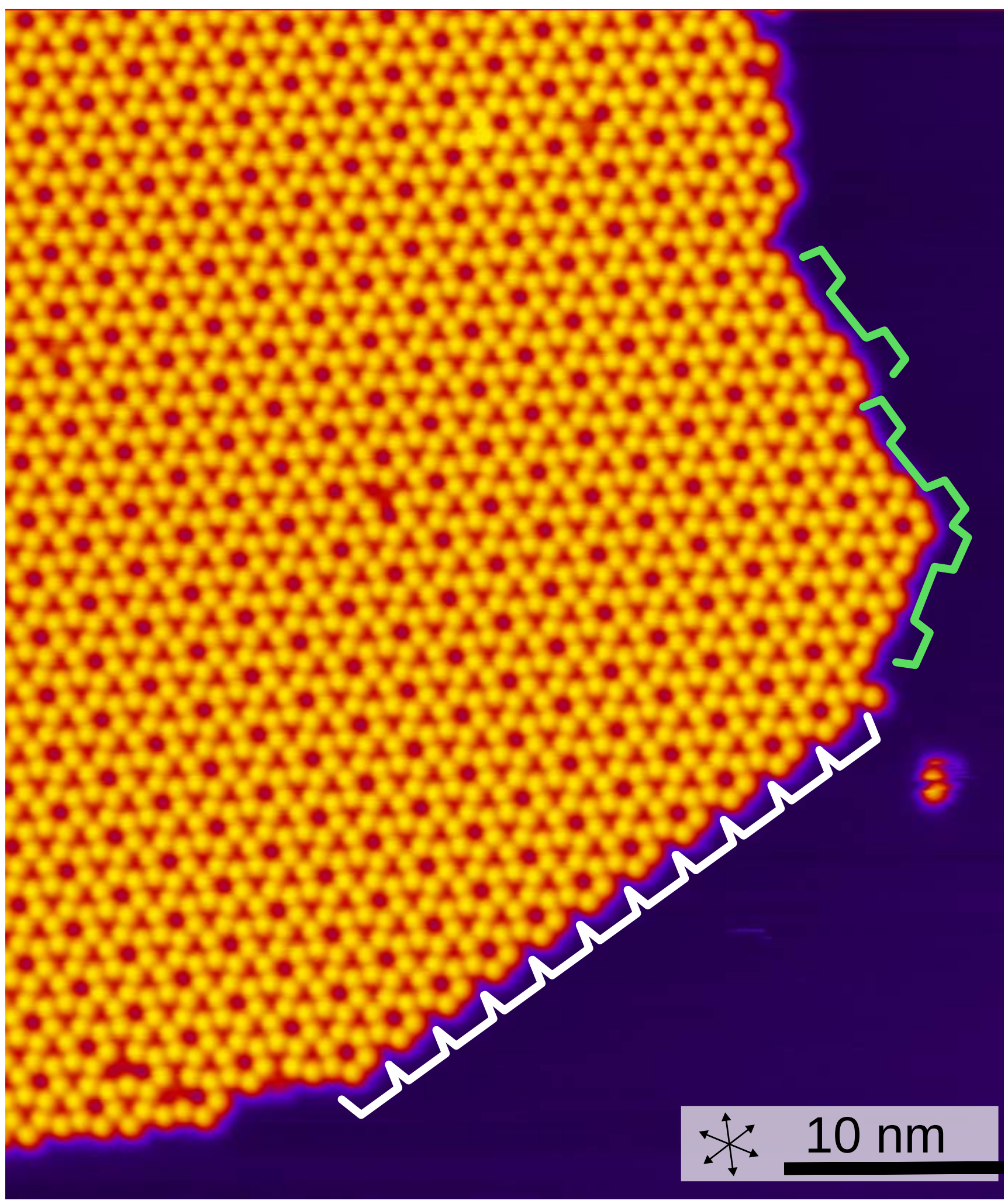

FIG. S1. Topograph of the edges of a 3-pyridyl-TOTA island. There is a tendency of island edges to align preferentially along the ⟨110⟩ (white line) and ⟨112⟩ (green lines) directions.

* shiotari@fhi-berlin.mpg.de
† roberto.robles@csic.es
‡ berndt@physik.uni-kiel.de

Ordering in Large 3-pyridyl-TOTA Islands

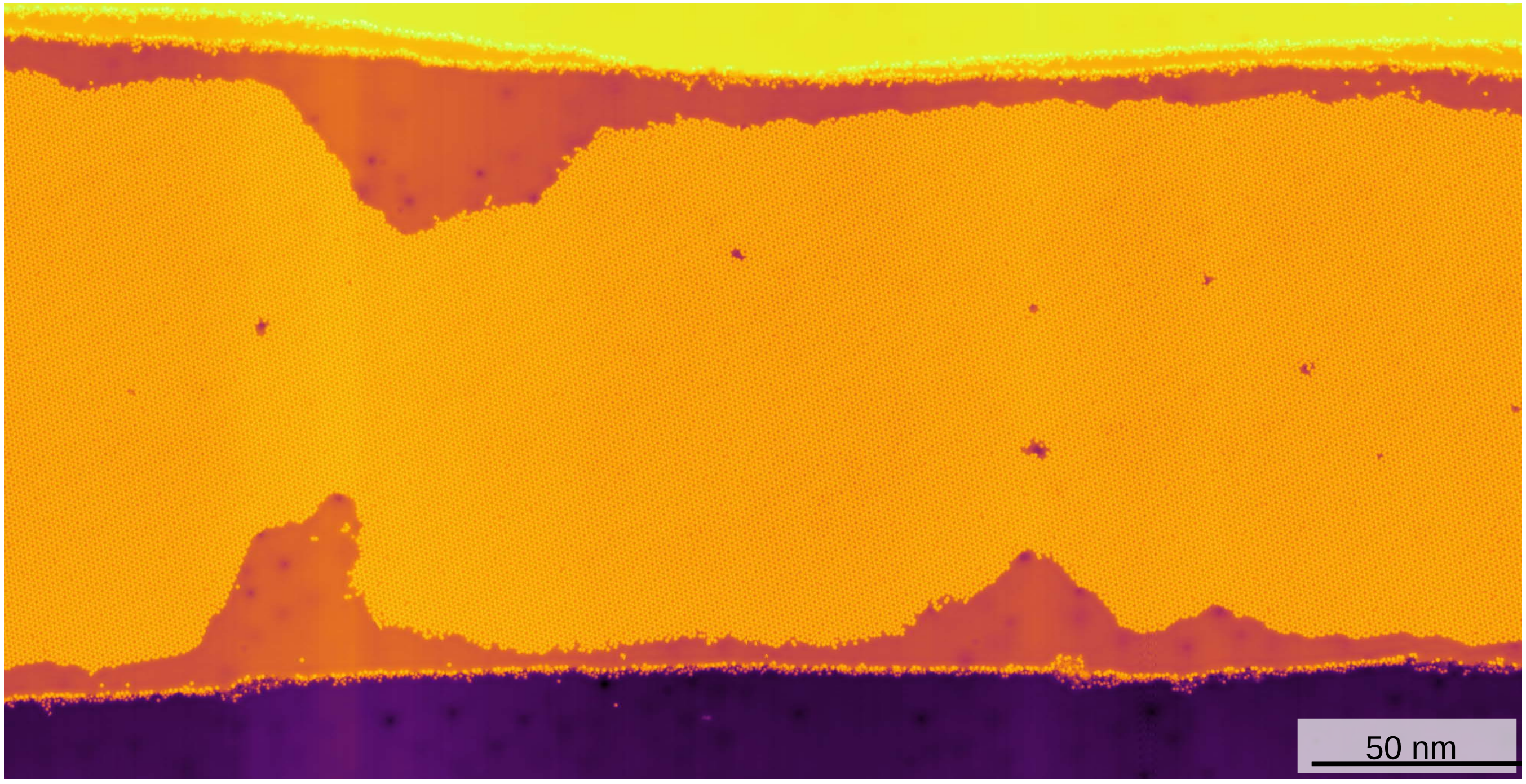

FIG. S2. Large island of 3-pyridyl-TOTA on Ag(111). The image sized 476×210 nm^2 shows a part of a large island. The number of the defects is small and the island is confined by Ag step edges at the top and bottom of the image. Similar islands were rather commonly observed.

Additional Spectroscopic Data on 3-pyridyl-TOTA

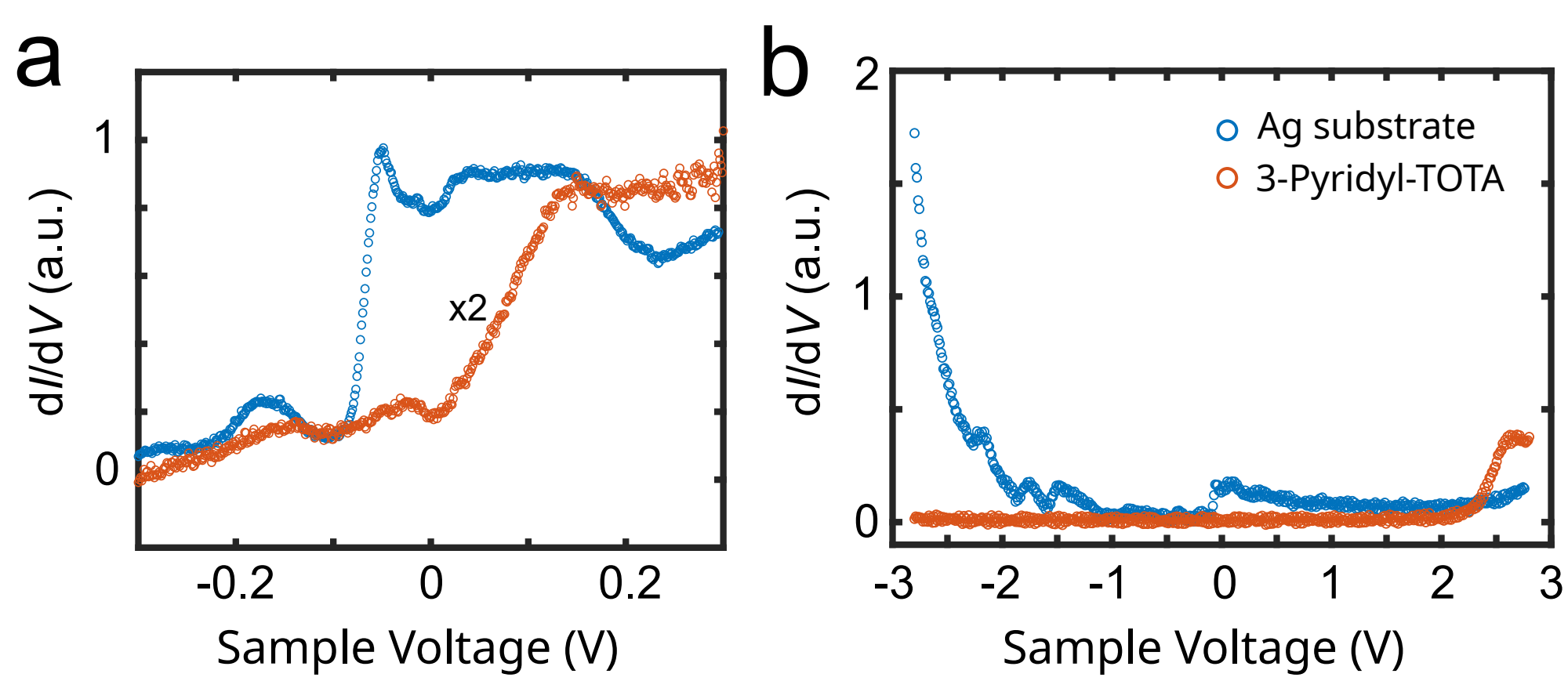

FIG. S3. Constant-height dI/dV spectra measured on 3-pyridyl-TOTA molecules in the spacious structure. (a) The Ag(111) surface state, which is signaled by a steep rise at ≈ -0.07 V (blue dots) is shifted under the molecular layer (red dots) to ≈ 0.85 V. (b) The spectrum of 3-pyridyl-TOTA on a wider voltage range, measured at larger tip height, is featureless except for the LUMO peak near 2.5 V, which is presented in the main text.

Hexagonally Close-Packed Islands

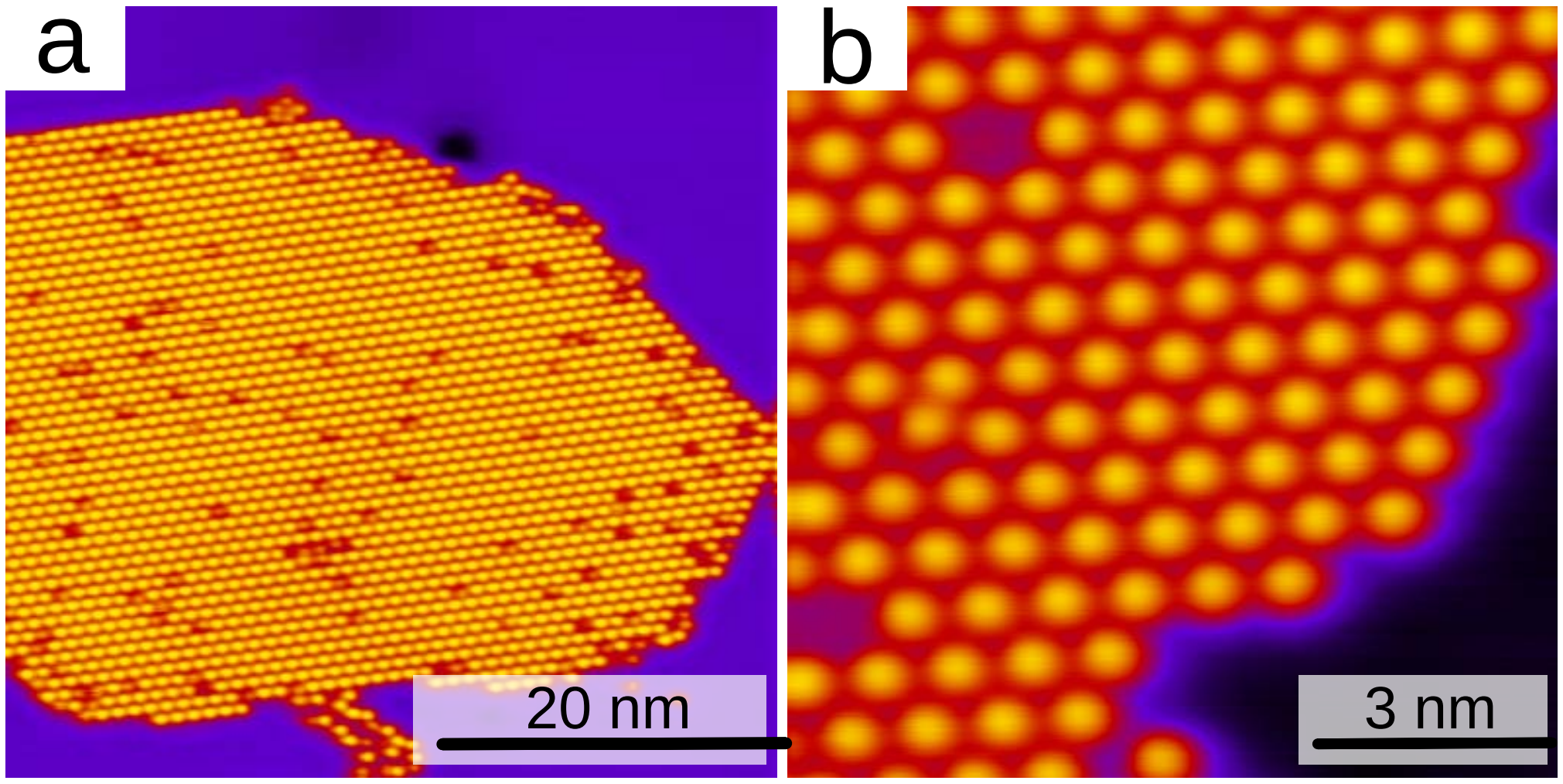

FIG. S4. (a) Overview topograph (0.13 V) of a hexagonally close-packed monolayer island on Ag(111). (b) More detailed topograph of an island of the same type. $V = 0.24\,\mathrm{V}$

Islands with a simple hexagonal pattern were occasionally observed (Figure S4). The nearest-neighbor distance $d \approx 1.04\,\mathrm{nm}$ is slightly larger in these islands than in the spacious hexagonal structure.

Figures S5(a) and (b) show STM and NC-AFM image, respectively, of a hexagonally closed-packed island measured with a CO tip. With NC-AFM, a 3-pyridyl-TOTA molecule in the island is imaged as a round bright spot with dark halo, similar to that in the spacious hexagonal island.

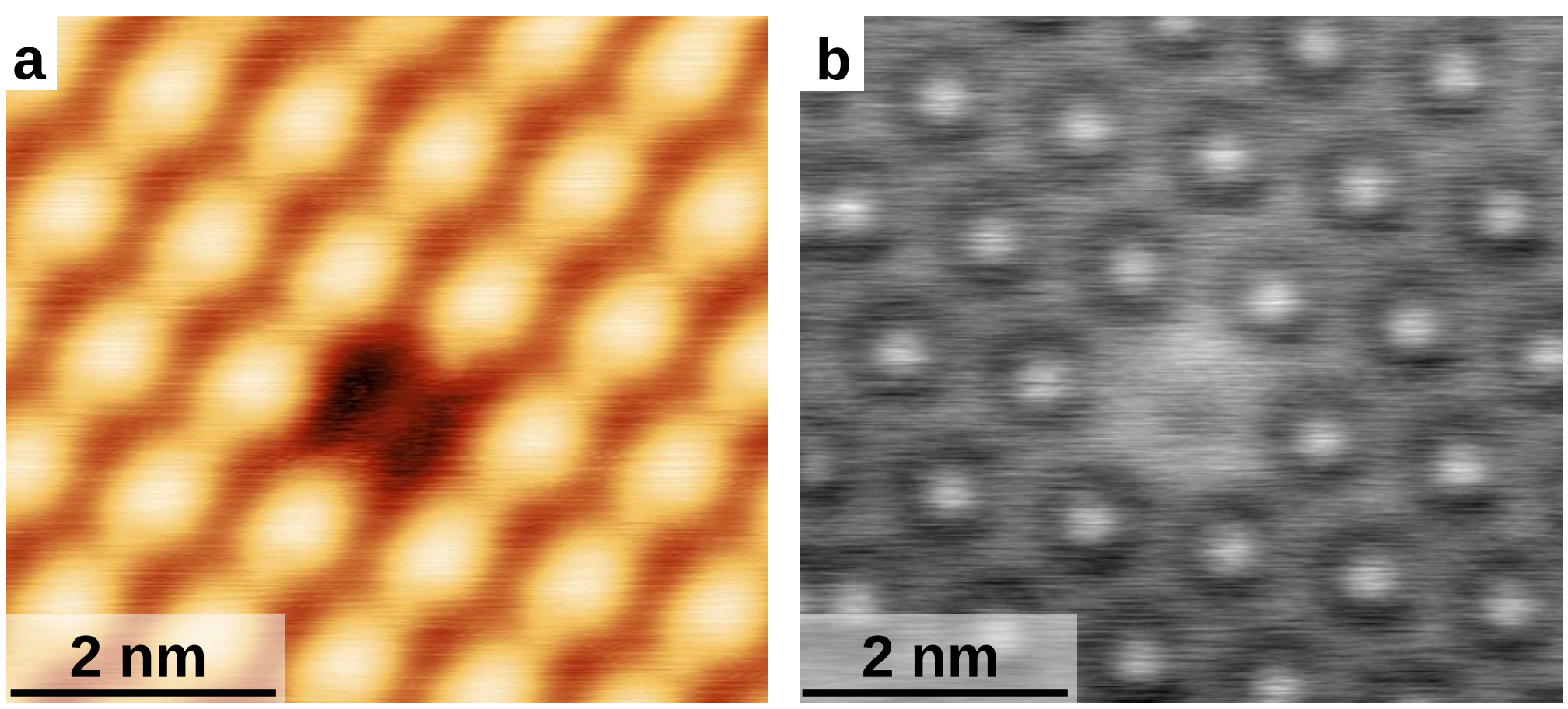

FIG. S5. (a) STM and (b) NC-AFM images of a closed-packed island with a vacancy using a CO tip. STM: constant current mode, 0.5 V, 20 pA. NC-AFM: constant height mode, $z = 25\,\mathrm{pm}$, $\Delta f = -13.1$ (black) to $-10.9\,\mathrm{Hz}$ (white).

Force Maps

As described in the main text, we obtained the background-subtracted $\Delta f(z)$ maps across the center of a 3-pyridyl-TOTA molecule in the x and y directions. Using the Sader–Javis formula [?], we obtain the maps of vertical force F_z acting to the tip, as shown in Figure S6. The positive and negative values represent the tip interacts attractively and repulsively with the molecule, respectively. The appearances of the F_z maps are very similar to the corresponding U maps shown in the main text.

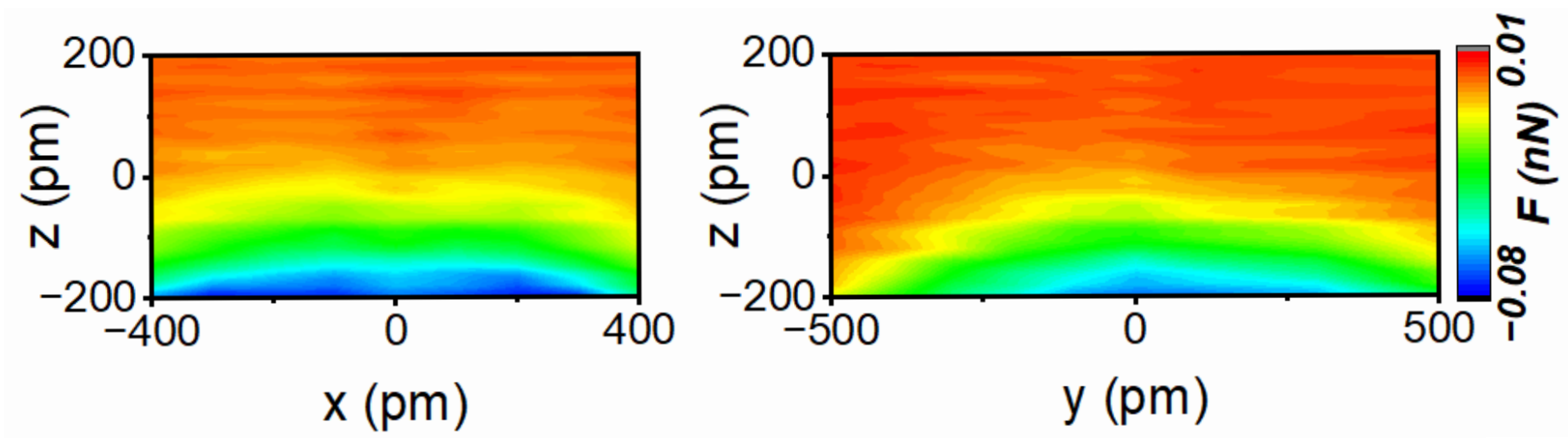

FIG. S6. Vertical force maps in the x–z plane at $y = 0$ (left) and in the y–z plane at $x = 0$ (right), obtained from the experimental Δf maps shown in the main text.

Method of Potential Energy Map Simulations

To simulate the U maps over the pyridyl-TOTA molecule, we calculated the potential energies between the metal tip and the molecule using the pairwise Leonard-Jones potential energies [?];

$$U_{\alpha,\beta} = \sqrt{\epsilon_\alpha \epsilon_\beta} \left[\left(\frac{r_\alpha + r_\beta}{r_{\alpha,\beta}} \right)^{12} - 2 \left(\frac{r_\alpha + r_\beta}{r_{\alpha,\beta}} \right)^{6} \right], \tag{1}$$

where $U_{\alpha,\beta}$ denotes the potential energy between atom α and atom β, and $r_{\alpha,\beta}$ is the distance between the two atoms. Table SI shows the element-dependent parameters of radius r_α and energy ϵ_α [?]. As atoms of the sample, we consider the pyridyl group and its anchoring C atom (C_6NH_4), as schematically shown in Figure S7. We approximated the tip structure as a single virtual atom, namely, probe particle (PP) [? ?].

TABLE SI. Parameters for the pairwise Leonard-Jones potential energies.

α	ϵ_α [meV]	r_α [Å]
C	3.729	1.908
N	7.372	1.824
H	0.68	1.478
PP	1000	2

Under the conditions, the potential energy U between the tip and molecule can be expressed as

$$U = \sum_{\alpha \in C_6NH_4} U_{\alpha,\mathrm{PP}}. \tag{2}$$

In the simulations, the tip–molecule distance d is defined as the vertical distance between the probe particle and the topmost H atom of the 3-pyridyl-TOTA molecule, *i.e.*, $r_{\mathrm{H_{top}},\mathrm{PP}} = \sqrt{x^2 + y^2 + d^2}$ at a tip position (x, y, d). By calculating U at each tip position, we obtained the $U(x, d)$ and $U(y, d)$ maps shown in the main text. We selected the displayed d range of the simulated maps (from 313 to 663 pm) where the map shapes and the corresponding line profiles closely matched those of the experiment ($z = -200$ to 200 pm; see the main text). Then $z = 0$ in the experiment, which is determined by the STM setpoint, corresponds to $d = 513$ pm. This distance is reasonable as a tunneling gap.

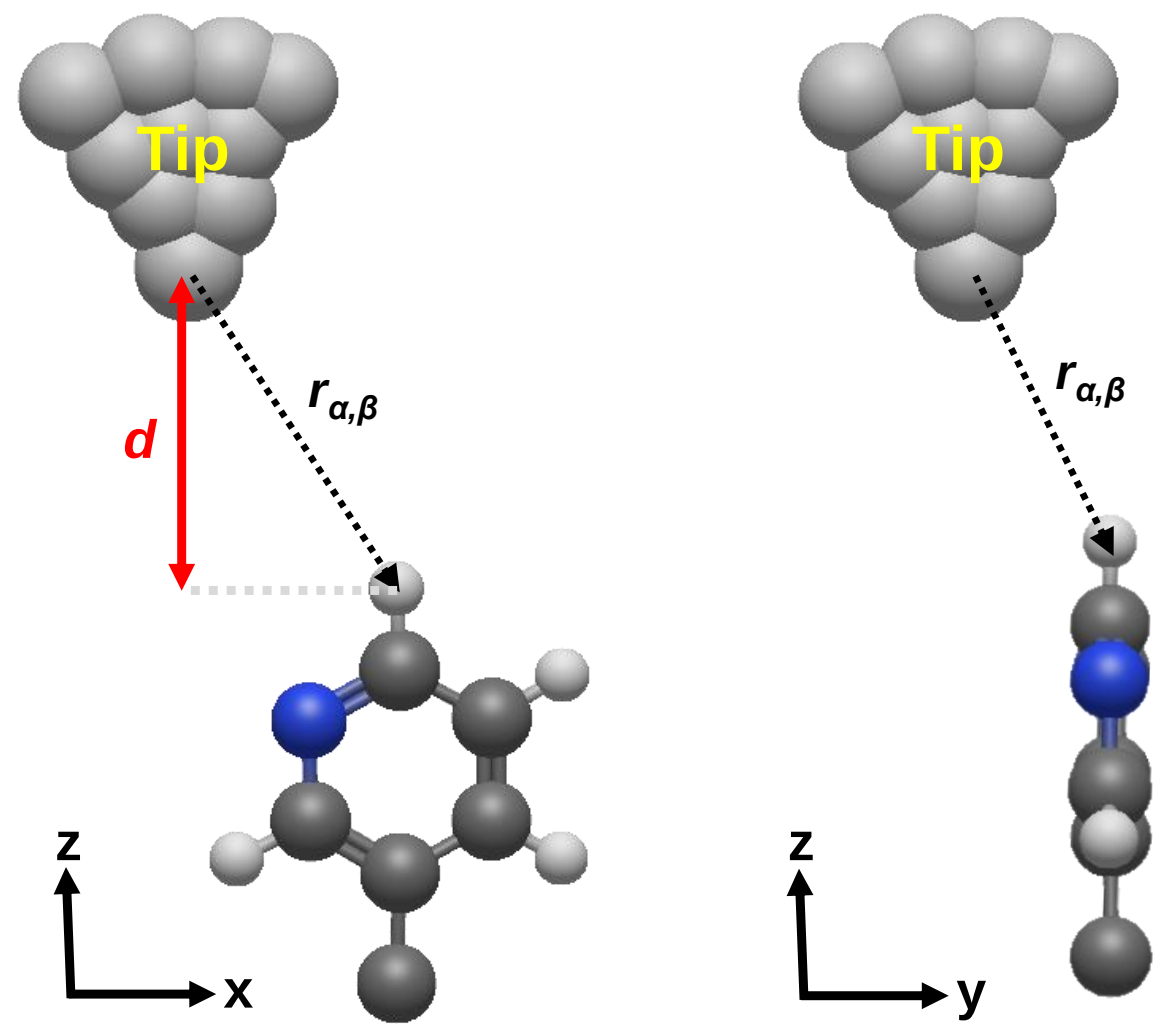

FIG. S7. Schematic atomic structure of the tip and 3-pyridyl-TOTA molecule. Black, blue, white, and gray spheres represent C, N, H, and Ag atoms, respectively. For the potential energy map simulations, the tip structure is approximated to be a PP.

Sec-Butyl-TOTA on Ag(111)

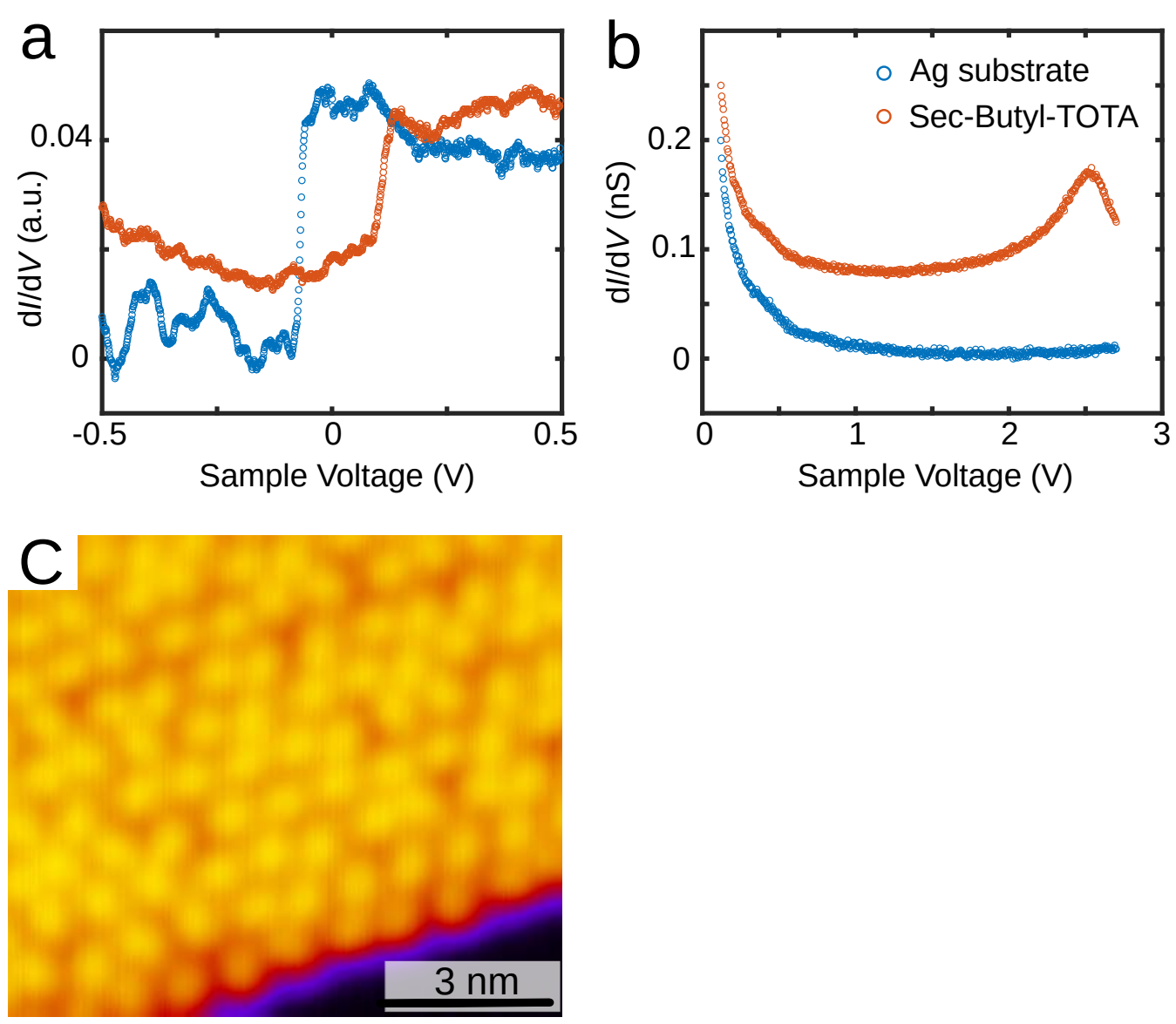

FIG. S8. dI/dV spectra of *sec*-butyl-TOTA and Ag(111). (a) The constant-height spectrum of Ag(111) spectrum (blue) shows the typical surface state onset near -70 mV. The molecular spectrum (red) exhibits a similar conductance rise at ≈ 0.11 V that we interpret as being due to the shifted surface state. The same shift was previously observed from phenyl-TOTA.[**?**] (b) Constant-current spectrum ($I = 20$ pA) revealing a resonance near $V = 2.5$ V. (c) Constant-current image measured at $V = 2.5$ V.

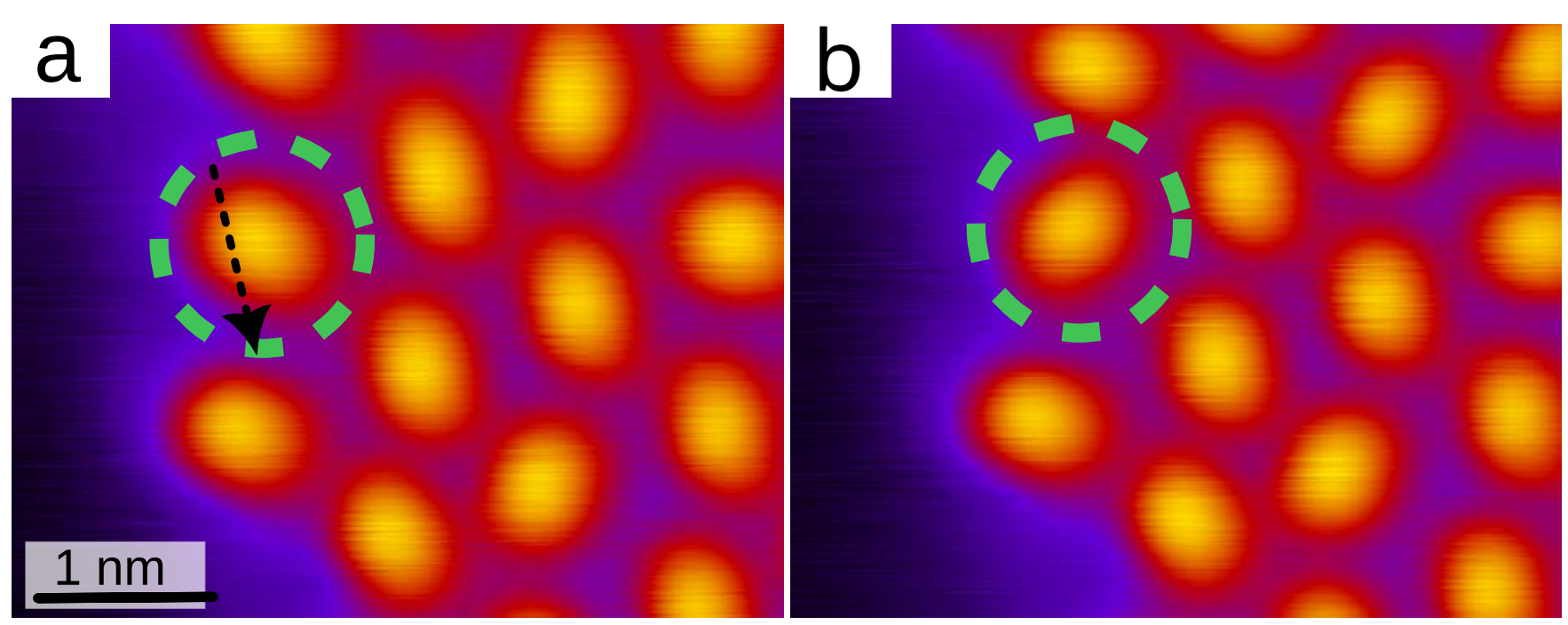

FIG. S9. Rotation of the *sec*-butyl ligand. (a) Topograph an edge of a *sec*-butyl-TOTA island on Ag(111). To induce a rotation of the ligand marked by a dashed circle, the STM tip was initially located near the tail of the black arrow and then brought closer to the surface (5 mV, 1.5 nA). Next, the tip was moved along the trajectory indicated by the arrow and then retracted to imaging conditions. (b) Image of the same area after the manipulation. The ligand is rotated.

Different Configurations of the Gas-Phase Dimer

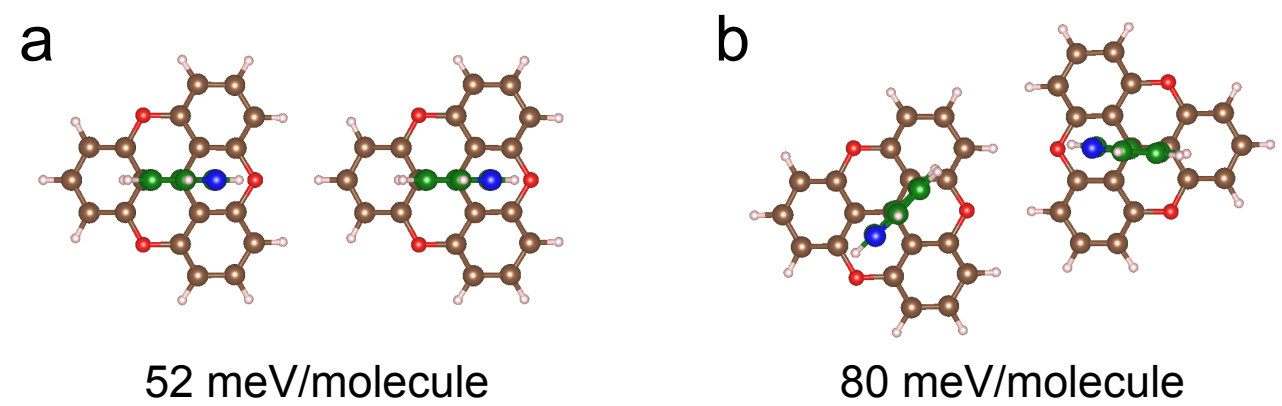

FIG. S10. (a) Corner-to-side and (b) side-to-side configurations of a molecular dimer in the gas phase and correspondingt binding energies. Brown, red, blue, and pink spheres represent C, O, N, and H atoms, respectively. Green spheres represent C atoms of the pyridyl moiety.

Figure S10 shows two configurations of the dimer. The corner-to-side configuration in (a) corresponds to the building motif of the close-packed layer. In this configuration, the molecules form one C-H $\cdots$ O hydrogen bond. The side-to-side configuration in (b) corresponds to the building motif of the spacious hexagonal layer. Here, the molecules form two C-H $\cdots$ O hydrogen bonds and consequently it is more stable by 28 meV/molecule.

Different Configurations of the Gas-Phase Layer

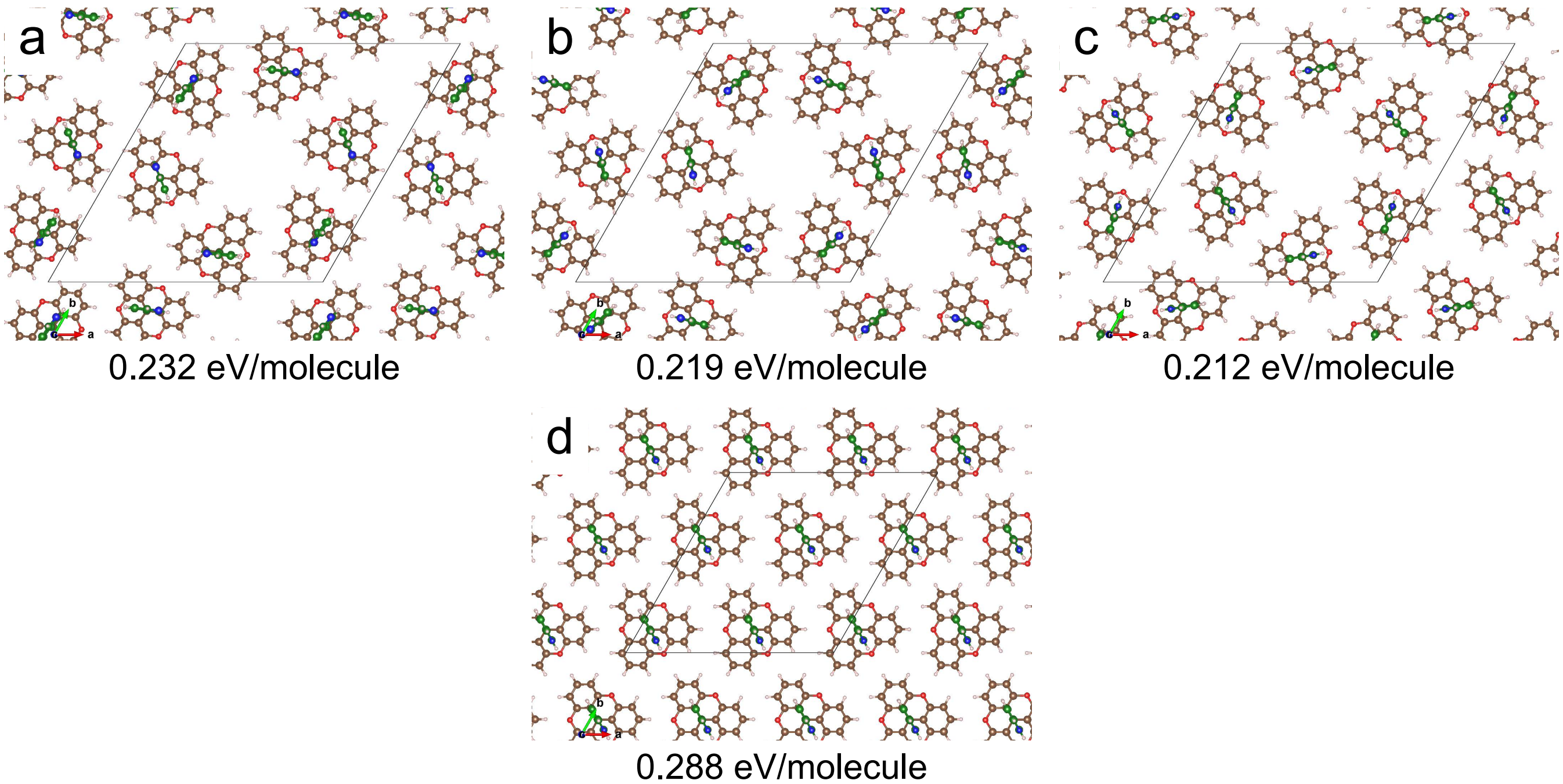

FIG. S11. Different configurations for the molecular layer in the gas phase and corresponding binding energies. (a – c) show configurations for the spacious hexagonal unit cell, while (d) shows a configuration for the hexagonal close-packed arrangement. Brown, red, blue, and pink spheres represent C, O, N, and H atoms, respectively. Green spheres represent C atoms of the pyridyl moiety. Black lines indicate the unit cell.

Figure S11 shows different configurations of the molecular layer in the gas phase, without substrate. Based on a hexagonal ring as the building block, panels (a – c) of Figure S11 correspond to three different configurations of the ring in the unit cell. Figure S11(a) matches the structure proposed from the experimental STM images and is the most stable among them. Figure S11(b) corresponds to a honeycomb lattice and it is 13 meV/molecule higher in energy. In the arrangement in Figure S11(c) the binding between hexagons is not side-to-side, but corner-to-side. This case is 20 meV/molecule higher in energy than the first one. Overall the comparison between these three different options confirms that the most stable option is the one observed experimentally. Figure S11(d) shows a layer in a close-packed configuration. This structure is 56 meV/molecule more favorable than the spacious hexagonal structure in Figure S11(a), similar to the case with substrate discussed in the main text.

Different Configurations of the Spacious Hexagonal Arrangement

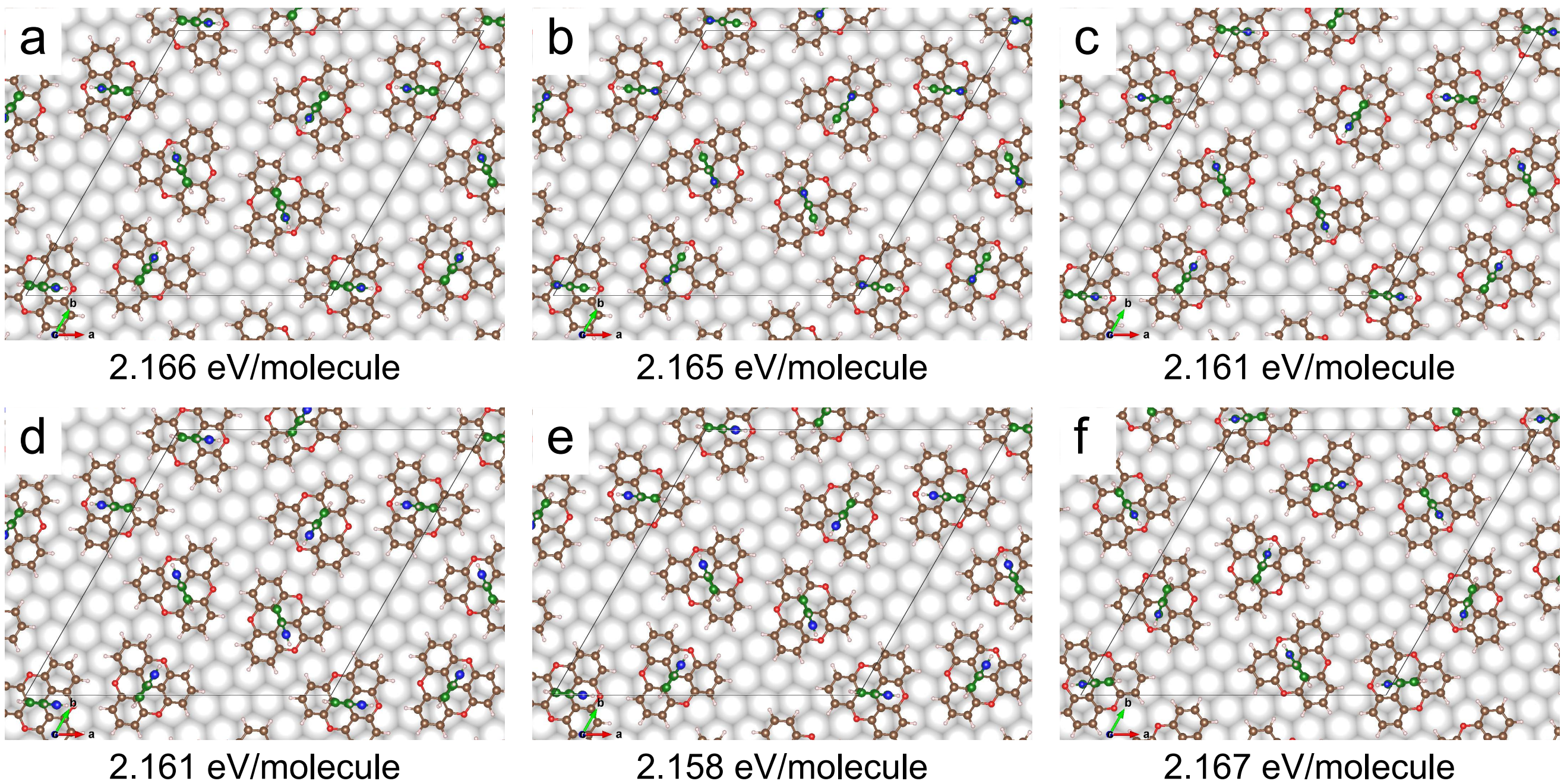

FIG. S12. Different configurations for the spacious hexagonal arrangement together with the corresponding adsorption energies. White, brown, red, blue, and pink spheres represent Ag, C, O, N, and H atoms, respectively. Green spheres represent C atoms of the pyridyl moiety. Black lines show the unit cell.

Figure S12 shows different configurations of the spacious hexagonal arrangement with different adsorption sites or orientations of the pyridyl groups. The unit cell is rotated by -5° with respect to the $\langle 1\bar{1}0\rangle$ direction. All of them are quite close in energy and differ by less than 10 meV per molecule, within the accuracy of the calculation. Figure S12(a) and (b) differ by the orientation of the pyridyl moieties and they are nearly degenerate. Figure S12(a) and (c–e) differ in the adsorption site of the motif on the Ag(111) surface. The small energy difference (within 8 meV/molecule) reflects a small preference for a particular adsorption site, which is consistent with an adsorption ruled by vdW forces. Finally, Figure S12(f) corresponds to a different chirality of the motif. In the calculation both chiralities i.e. (a) and (f) are nearly degenerate. In the gas phase both chiralities have the exact same energy. When adsorbed they differ in the orientation of the molecules with respect to the surface. For the first chirality (Figure S12(a–e)) all the molecules have a side forming $\approx 20^\circ$ with a $\langle 1\bar{1}0\rangle$ direction of the surface. However, for the second chirality in (f) one side of all the molecules is oriented along a $\langle 11\bar{2}\rangle$ direction. Experimentally, for the unit cell studied here having a -5° angle, only the chirality in (a–e) is observed. This indicates an experimental preference for the molecules to form $\approx 20^\circ$ with the $\langle 1\bar{1}0\rangle$ direction. This preference is not captured by the present theoretical approach, for which both options are nearly degenerate.

Single 3-pyridyl-TOTA Adsorbed on Ag(111)

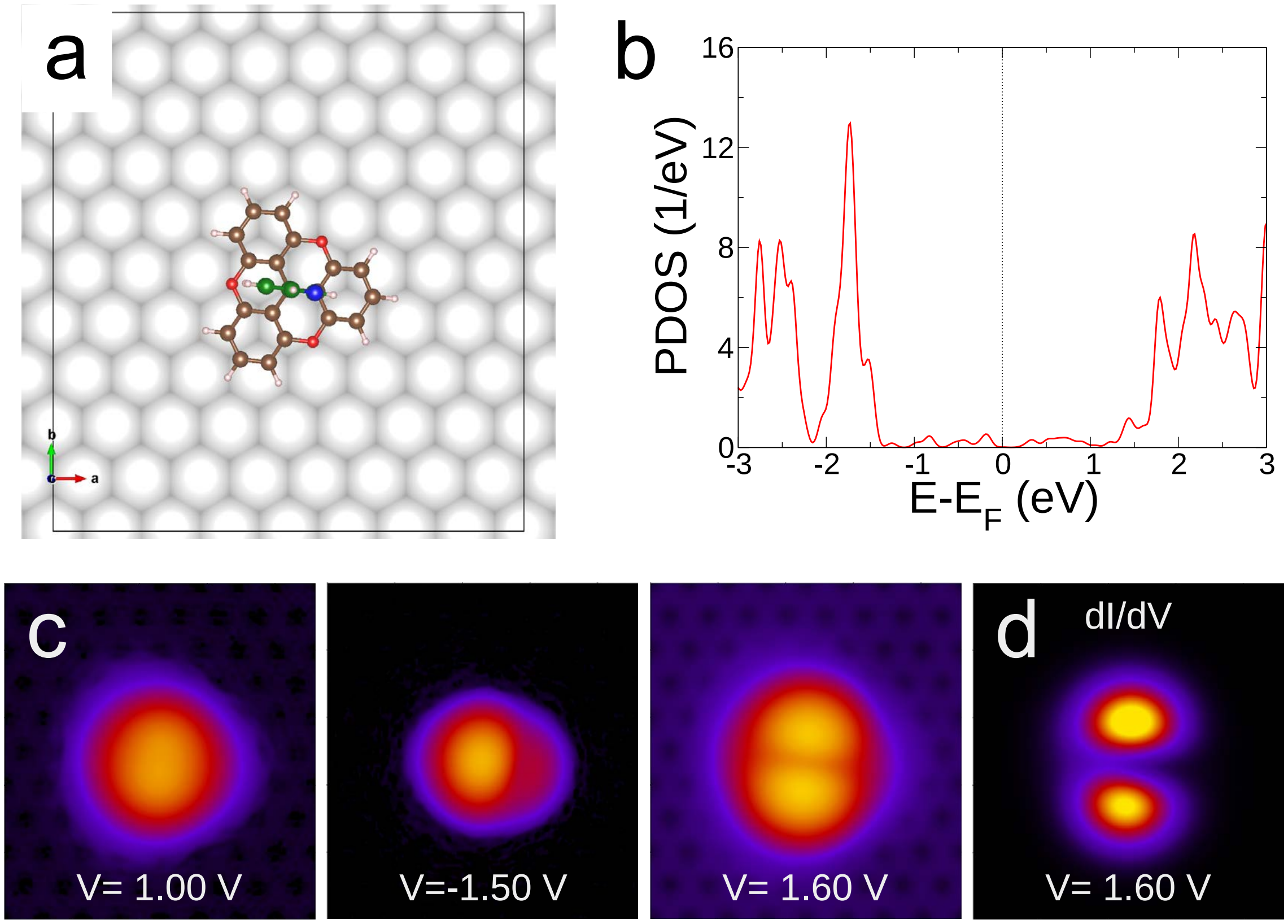

FIG. S13. (a) Geometry of one 3-pyridyl-TOTA adsorbed on Ag(111). White, brown, red, blue, and pink spheres represent Ag, C, O, N, and H atoms, respectively. Green spheres represent C atoms of the pyridyl moiety. Black lines show the unit cell. (b) Density of states projected on the molecule. (c) Simulated constant current STM images calculated at the indicated voltage. (d) Simulated dI/dV map at $V = 1.6$ V.

Figure S13 shows one 3-pyridyl-TOTA adsorbed on Ag(111). The theoretical details are as indicated in the main text but here a rectangular (8×5) in-plane unit cell is used. The PDOS in Figure S13(b) presents the same features as for the spacious hexagonal arrangement show in the main text. The binding energy in this case is 2.063 eV. Figure S13(c) shows simulated constant current topographs. The results are similar to the ones shown in the text for the spacious hexagonal arrangement: within the molecular energy gap ($V =$ 1.00 and -1.50 V) the molecule shows a round aspect; on the onset of the first unoccupied state ($V =$ 1.60 V) a node starts to be visible. Figure S13(d) shows a simulated dI/dV map at $V = 1.60$ V. It resembles the "arrows" observed experimentally for the array.

Origin of the "arrow" shape

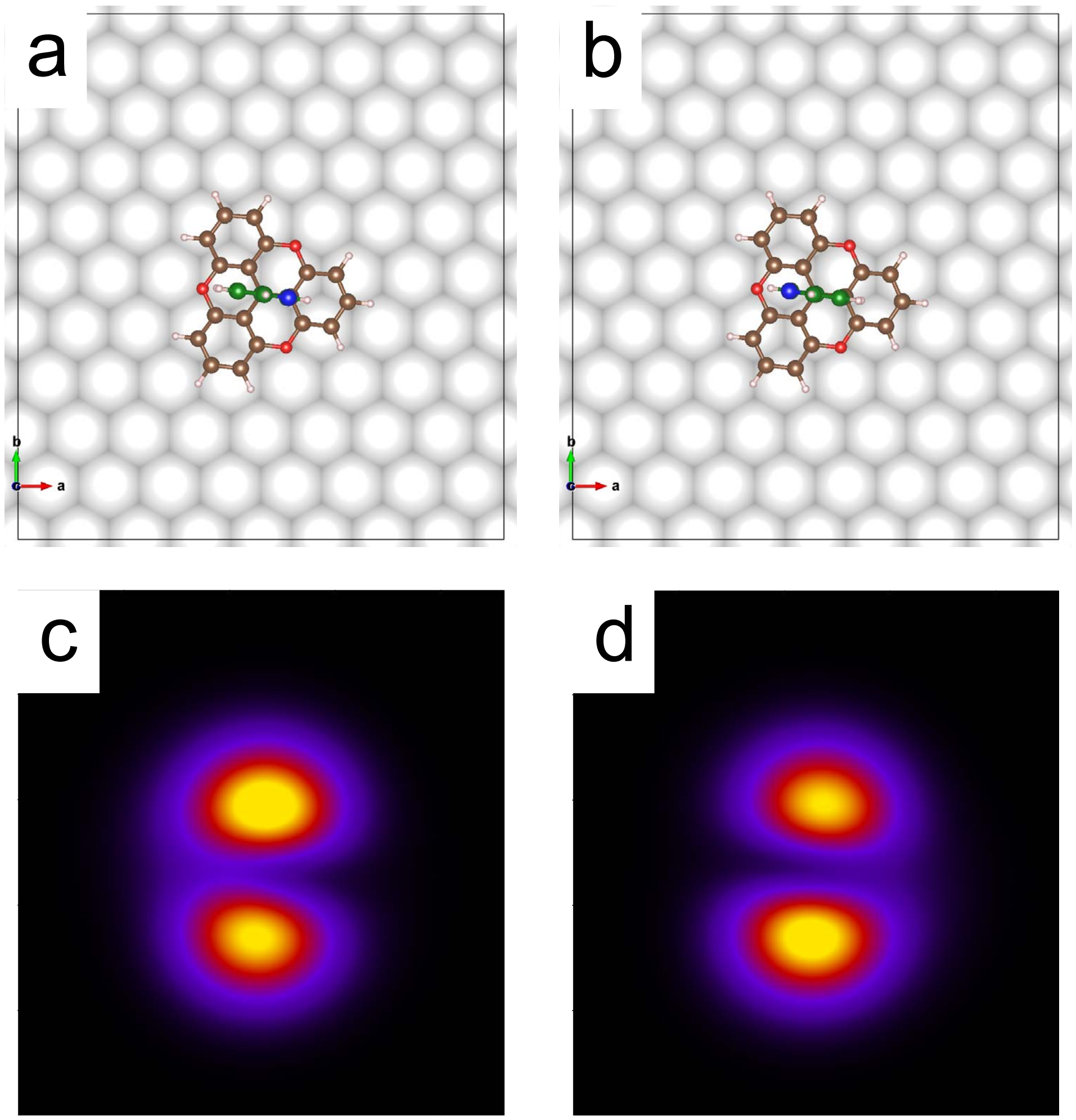

FIG. S14. Geometry of one 3-pyridyl-TOTA adsorbed on Ag(111) with the N atom of the pyridyl group pointing to the right (a) or to the left (b). White, brown, red, blue, and pink spheres represent Ag, C, O, N, and H atoms, respectively. Green spheres represent C atoms of the pyridyl moiety. Black lines show the unit cell. (c–d) Calculated constant-height dI/dV at $V = 1.6\,\mathrm{V}$ for the configurations in (a–b).

In order to establish the origin of the "arrow" shape observed experimentally, we have compared two 3-pyridyl-TOTA molecules with the same adsorption position and orientation. One of them has the N atom of the pyridyl group pointing to the right (Figure S14a), while in the other it is pointing to the left, i.e., towards one O atom of the TOTA platform Figure S14b). The comparison of the simulated constant-height dI/dV maps at $V = 1.6\,\mathrm{V}$ in (Figure S14(c–d) shows that the origin of the arrow shape is indeed the orientation of the pyridyl group. The open end of the arrow corresponds to the location of the N atom. Therefore, by looking at the experimental shape of the dI/dV maps at the onset of the first unoccupied peak, we are able to completely determine the position and orientation of the pyridyl moieties.

Synthesis of 12c-(Pyridin-3-yl)-4,8,12-trioxatriangulene

Under a nitrogen atmosphere, a 1.3 M solution of isopropylmagnesium chloride lithium chloride complex (*i*-PrMgCl · LiCl) in tetrahydrofuran (THF) (0.5 mL, 650 μmol) was added dropwise to a solution of 3-iodopyridine (140 mg, 683 μmol) in anhydrous THF (5 mL), and the mixture was stirred at room temperature for 45 min. The resulting solution was then added, under a nitrogen atmosphere, to a suspension of 4,8,12-trioxatriangulenium tetrafluoroborate salt (150 mg, 403 μmol) in anhydrous THF (5 mL) and heated under reflux for 18 h. Subsequently, the solvent was removed *in vacuo*, and the crude product was purified by column chromatography on basic aluminum oxide (cyclohexane/ethyl acetate, 3:1, $R_f = 0.51$). A pale brown solid (8 mg 22.0 μmol, 5%) was obtained.

Melting point: 228 °C

^{1}H-NMR (600 MHz, Aceton-d6, 300 K, TMS): $\delta = 8.38 - 8.35$ (m, 1H, *H*-6), 8.26 – 8.23 (m, 1H, *H*-2), 7.46 (t, ^{3}J = 8.2Hz, 3H, *para-*H_{TOTA}), 7.37 - 7.33 (m, 1H, *H*-4), 7.27-7.23 (m, 1H, *H*-5), 7.12 (d, 3J = 8.2Hz, 6H, *meta-*H_{TOTA}) ppm.

^{13}C-NMR (150 MHz, Aceton-d6, 300 K): $\delta = 153.4$ (*ortho-*C_{TOTA}), 149.7 (*C*-6), 147.9 (*C*-2), 142.4 (*C*-3), 133.7 (C-4), 130.6 (*para-*C_{TOTA}), 124.8 (*C*-5), 113.9 (*ipso-*C_{TOTA}), 112.8 (*meta-*C_{TOTA}), 31.6 (*center-*C_{TOTA}) ppm.

MS (MALDI-TOF): m/z (%) =364.2 (43) $[M+H]^+$, 285.3 (100) $[TOTA]^+$.

HR-MS (ESI): m/z = calc. 364.09682 for $C_{24}H_{14}O_3N$, found 364.09621 ($\Delta = -1.68$ ppm).

IR (ATR): $\tilde{v} = 2928$ (w), 2857 (w), 1714 (w), 1610 (s), 1479 (m), 1455 (s), 1419 (w), 1262 (s), 1134 (m), 1065 (m), 1012 (s), 912 (m), 784 (s), 757 (m), 736 (m), 709 (m), 665 (m), 624 (w), 582 (m), 559 (w), 518 (w), 507 (w) cm^{-1}.

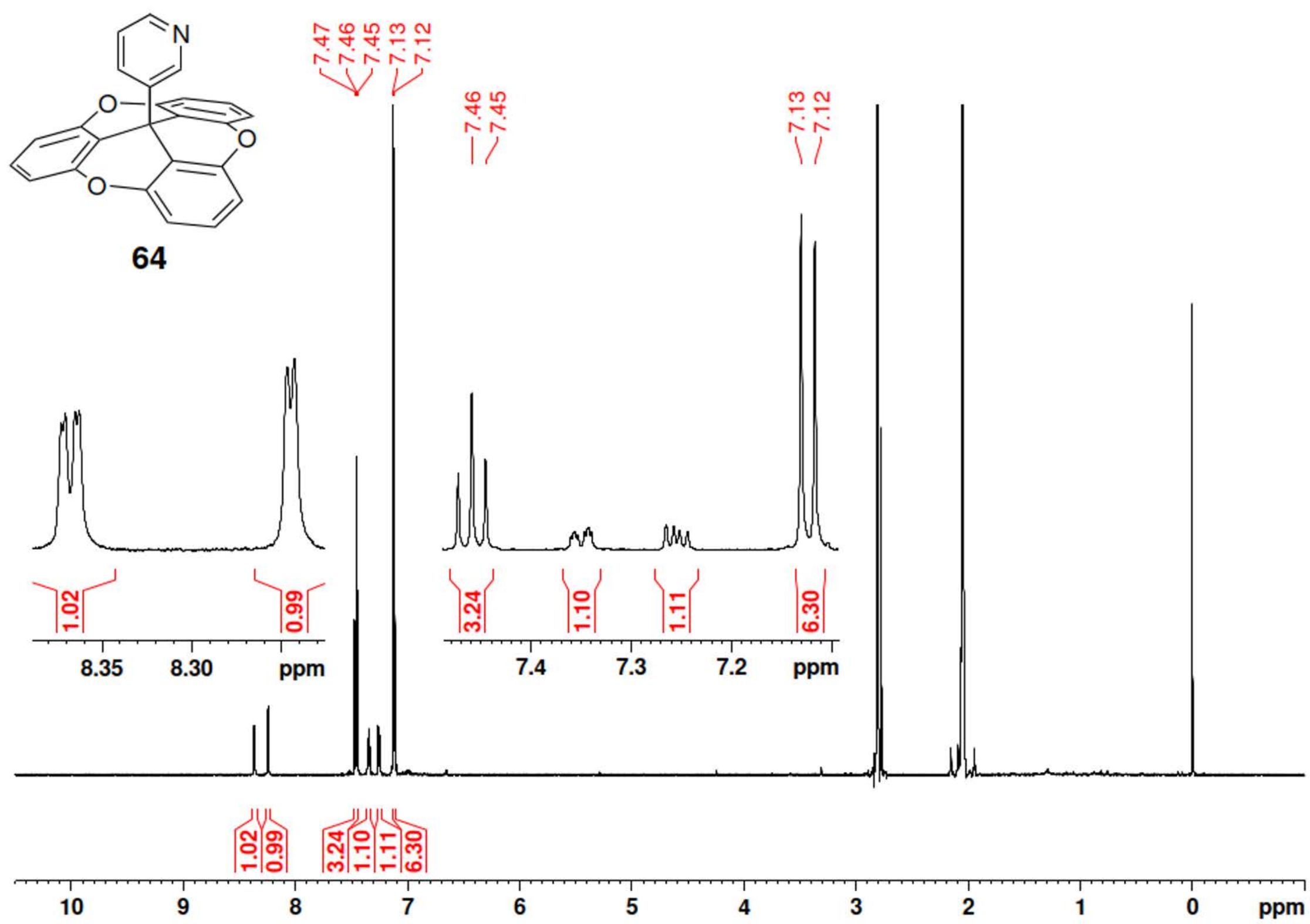

FIG. S15. 1H-NMR spectrum (600 MHz) of 12c-(pyridine-3-yl)-4,8,12-trioxatriangulene.

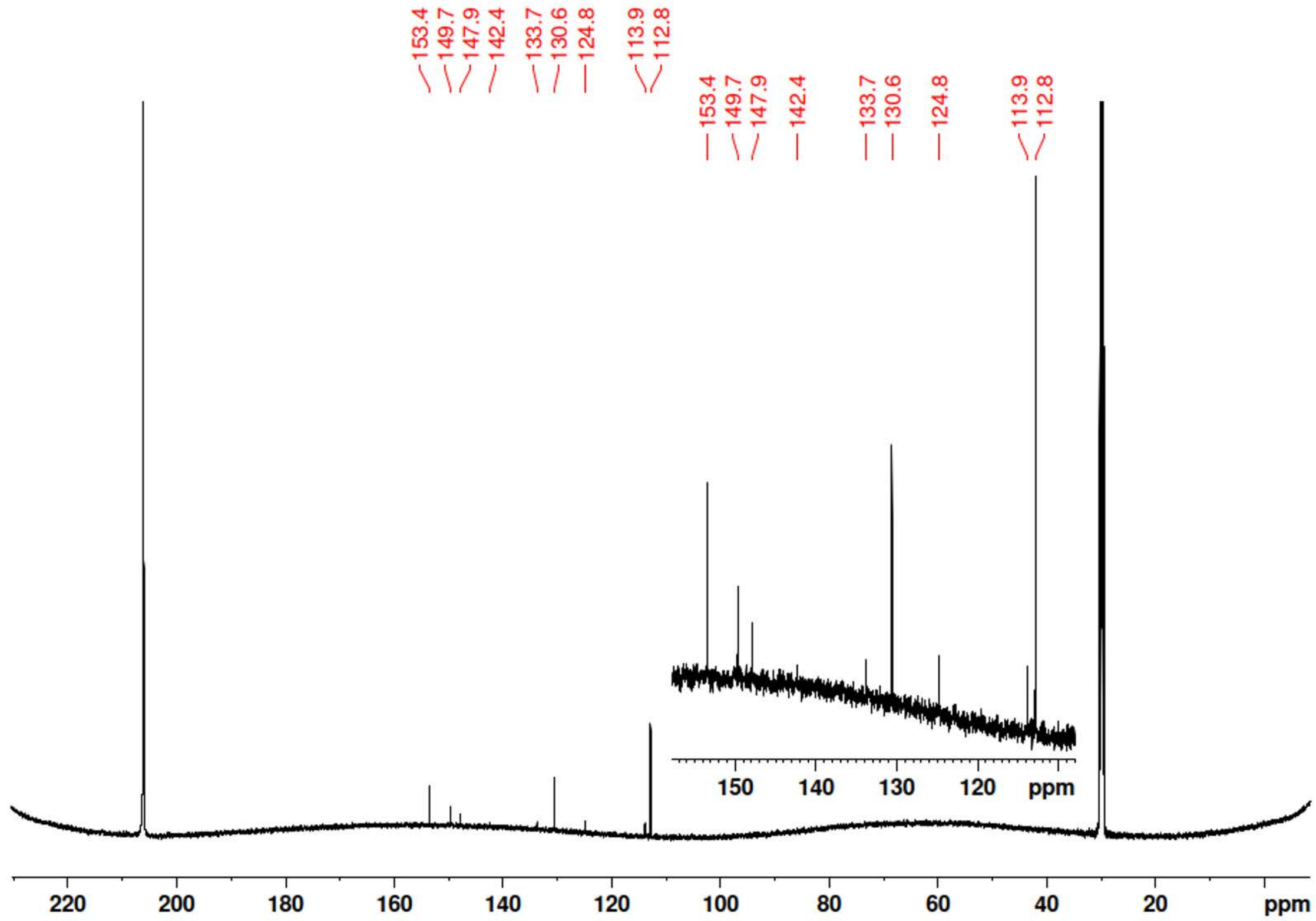

FIG. S16. ^{13}C-NMR spectrum (150 MHz) of 12c-(pyridine-3-yl)-4,8,12-trioxatriangulene.
